# SARS-CoV-2 ORF3a suppresses host antiviral interferon responses by promoting STUB1-mediated PTEN proteasomal degradation

**DOI:** 10.1128/jvi.00186-26

**Published:** 2026-06-02

**Authors:** Lujie Fan, Xiang Gao, Wei Feng, Qiang Huang, Xiafei Wei, Chuwei Yang, Yezi Wu, Xiaotong Shen, Juanjuan Zhao, Yuzheng Zhou, Zheng Zhang

**Affiliations:** 1 Institute for Hepatology, National Clinical Research Center for Infectious Disease, Shenzhen Third People’s Hospital, Department of Biochemistry, the Second Affiliated Hospital, School of Medicine, Southern University of Science and Technology, Shenzhen, Guangdong, China; 2 Guangdong Key Laboratory for Anti-Infection Drug Quality Evaluation, Shenzhen, Guangdong, China; 3 Shenzhen Research Center for Communicable Disease Diagnosis, Treatment of Chinese Academy of Medical Science, Shenzhen, Guangdong, China; University of Michigan Medical School, Ann Arbor, Michigan, USA

**Keywords:** PTEN, STUB1, ORF3a, ubiquitination, degradation

## Abstract

Human coronaviruses (HCoVs) are a group of RNA viruses characterized by high genetic variability and cross-species transmission potential. They can cause a wide spectrum of respiratory illnesses, ranging from mild upper respiratory tract infections to severe pneumonia, and acute respiratory distress syndrome. PTEN, a well-known tumor suppressor, not only plays a crucial role in tumorigenesis but also enhances antiviral immunity by regulating IRF3 phosphorylation and promoting type I interferon production. In this study, we observed that PTEN significantly inhibited the replication of various HCoVs, including SARS-CoV-2, HCoV-229E, and HCoV-OC43. However, SARS-CoV-2 infection antagonizes the antiviral function of PTEN. Mechanistically, PTEN undergoes ubiquitination at lysine 6, followed by proteasomal degradation after SARS-CoV-2 infection. Through screening, it was found that STUB1 is the key E3 ligase responsible for PTEN degradation under SARS-CoV-2 infection. Furthermore, screening of SARS-CoV-2-encoded proteins revealed that ORF3a promotes STUB1-mediated PTEN ubiquitination and degradation at K6. Previous studies have shown that Oroxin B can exert the effect of a PTEN agonist by upregulating the expression of PTEN. In this study, we demonstrated that Oroxin B significantly enhances antiviral responses in mice. In conclusion, this study reveals the molecular mechanism by which SARS-CoV-2 evades PTEN-mediated antiviral effects, providing new insights for the development of PTEN-targeted antiviral strategies.

**IMPORTANCE:**

Human coronaviruses continue to threaten global health, yet how these viruses evade our natural antiviral defenses remains poorly understood. This study reveals that PTEN, a well-known tumor suppressor, also acts as a powerful antiviral molecule capable of limiting multiple human coronaviruses, including SARS-CoV-2. We further show that SARS-CoV-2 dismantles this protection by triggering PTEN degradation through the host enzyme STUB1 and the viral protein ORF3a. This discovery uncovers a previously unknown strategy used by coronaviruses to weaken innate immunity. Importantly, we identify Oroxin B as a promising compound that enhances antiviral responses *in vivo*. Together, our findings provide new insight into how coronaviruses disarm host defenses and highlight PTEN as a potential target for broad-spectrum antiviral therapies.

## INTRODUCTION

Human coronaviruses (HCoVs) are enveloped, positive-sense single-stranded RNA viruses (1, 2). Based on their pathogenic potential, HCoVs are broadly categorized into highly pathogenic strains, including SARS-CoV, MERS-CoV, and SARS-CoV-2, and low-pathogenic strains such as HCoV-229E, HCoV-OC43, HCoV-NL63, and HCoV-HKU1 (3). While highly pathogenic HCoVs are known to cause severe lower respiratory tract infections and acute respiratory distress syndrome (ARDS) (4, 5), low-pathogenic strains generally lead to mild upper respiratory symptoms similar to those of the common cold. However, in vulnerable populations, including infants, the elderly, and immunocompromised individuals, even these low-pathogenic viruses can lead to serious lower respiratory tract illnesses (6, 7). Given their seasonal circulation and widespread prevalence, low-pathogenic HCoVs represent a persistent global health burden (8, 9). Together with emerging pathogens like SARS-CoV-2, these viruses underscore the urgent need for broad-spectrum antiviral strategies.

SARS-CoV-2, the virus responsible for the COVID-19 pandemic, has evolved multiple mechanisms to suppress host innate immune responses, particularly the type I interferon (IFN-I) pathway. Several viral proteins, such as ORF6 (10–12), NSP1 (13–15), and ORF3a (16–18), have been shown to suppress IFN production or downstream signaling by targeting critical components such as IRF3 (19), STAT1 (20), and MAVS (21). These evasion strategies enable the virus to delay host immune activation and establish productive infection. Despite recent advances, our understanding of the host cell signaling pathways that are manipulated by SARS-CoV-2 to circumvent antiviral immunity remains incomplete.

PTEN (phosphatase and tensin homolog), widely recognized for its tumor-suppressive functions, has also emerged as a key regulator of antiviral immunity (22, 23). In addition to its classical function in restraining the PI3K/AKT/mTOR pathway (24), PTEN contributes to innate immune activation by enhancing IRF3 signaling and promoting IFN-I production (25). Despite this emerging role, it remains unclear how PTEN is modulated during coronavirus infection or whether it is actively targeted by viral proteins to promote immune evasion and viral replication.

Post-translational modifications (PTMs) play pivotal roles in regulating host cellular responses and viral life cycles during infection (26). Among them, ubiquitination, a reversible covalent modification involving the binding of ubiquitin to a protein, is a key mechanism to regulate protein function (27). Ubiquitination governs a wide array of cellular processes, including signal transduction, immune responses, intracellular trafficking, and protein degradation, many of which are actively manipulated by viruses to promote their replication and evade host immunity (28–32). In order to resist the host immune response, viruses hijack the host ubiquitin-proteasome system (UPS), either by encoding viral E3 ubiquitin ligases or by co-opting host enzymes, to selectively degrade antiviral proteins such as interferon signaling components (e.g., IRF3, STAT1) or to stabilize viral proteins (20, 33–35). Furthermore, ubiquitination also regulates the activation of innate immune sensors, including RIG-I and STING, thus determining the strength and duration of antiviral responses (36–39). Conversely, some viruses interfere with deubiquitinating enzymes (DUBs) to counteract host defense pathways (31, 40–44). Thus, a complex reciprocal game is played between the host and the virus by regulating the ubiquitination system.

This study examines the role of PTEN in host defense against HCoV infection and reveals a previously unknown strategy by which SARS-CoV-2-encoded ORF3a-mediated STUB1 promotes PTEN degradation to evade the host’s antiviral immunity. Our findings suggest that PTEN has a broad spectrum of antiviral activity against a variety of HCoVs and that SARS-CoV-2 promotes its disassembly by utilizing STUB1. Together, these findings not only confirm PTEN as a neglected but important antiviral factor in host protection against multiple HCoVs but also highlight its promise as a therapeutic target for the development of broad-spectrum coronavirus interventions.

## RESULTS

### PTEN enhances IFN-I responses and inhibits HCoV replication

PTEN is known as a tumor suppressor due to its regulatory role in the PI3K-AKT signaling pathway, and PTEN has been reported to enhance IFN-I responses by removing inhibitory phosphorylation on IRF3, thereby contributing to antiviral immunity. However, its precise role during HCoV infection remains poorly defined. In this study, we used Huh7 (a human hepatoma cell line) cells permissive to multiple HCoV infections to investigate the role of PTEN in antiviral signaling. PTEN was either overexpressed or silenced using shRNA, followed by infection with Sendai virus (SeV), a well-established model for stimulating RIG-I-like receptor-mediated IFN signaling.

**Fig 1:**
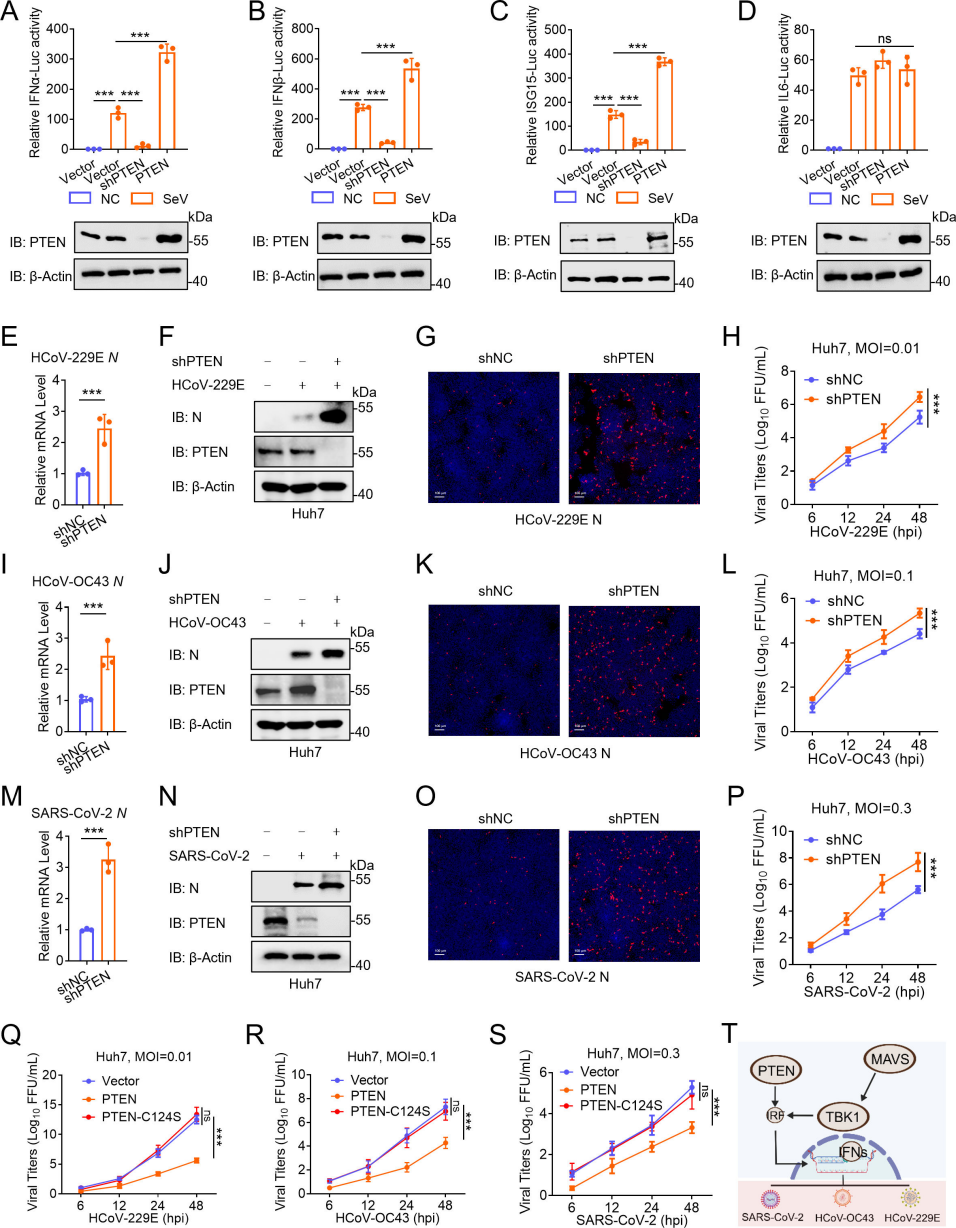
PTEN increases IFNs to inhibit HCoV replication. (**A–D**) Huh7 cells were co-transfected with luciferase reporter constructs driven by the promoters of IFNα, IFNβ, ISG15, and IL6, alongside the RL-TK vector serving as an internal normalization control. After 24 h, cells were infected with SeV at a multiplicity of infection (MOI) of 1 and incubated for an additional 24 h. Luciferase activity assays were then performed on cell lysates to evaluate promoter activation. (**E–P**) After PTEN knockdown by shRNA in Huh7 cells, HCoV-229E (MOI = 0.01), HCoV-OC43 (MOI = 0.1), or SARS-CoV-2 (MOI = 0.3) was used for infection. At 24 h after SARS-CoV-2 infection, the expression of viral *N* gene was detected by RT-qPCR (**E, I, M**), the expression of viral N protein was detected by Western blotting (**F, J, N**), and the level of viral N protein was observed by immunofluorescence staining (**G, K, O**). Culture supernatants were collected at multiple time points post-infection (6, 12, 24, and 48 h) and used to infect Vero E6 cells for measurement of infectious viral titers (**H, L, P**). (**Q–S**) Huh7 cells overexpressing PTEN or PTEN-C124S were infected with HCoV-229E, HCoV-OC43, or SARS-CoV-2, and supernatants collected at the indicated time points were titrated on Vero E6 cells to quantify infectious virus production (6, 12, 24, and 48 hpi). (**T**) A schematic diagram illustrates the role of PTEN in enhancing IFN-I responses and inhibiting coronavirus replication. Data are expressed as mean ± SD from a minimum of three independent assays. Statistical significance was determined using an unpaired Student’s *t*-test, with ns indicating *P* > 0.05, *P < 0.05, **P < 0.01, and ***P < 0.001.

Overexpression of PTEN significantly enhanced IFN-I (IFNα and IFNβ) and ISG15 (interferon-stimulated gene 15) without altering inflammatory cytokines (IL-6 and CXCL1), while knockdown of PTEN significantly inhibited IFN-I (Fig. 1A through D; Fig. S1A). To rule out confounding effects of SeV infection, cells were stimulated with the N-terminal domain of RIG-I (RIG-I-N), which recapitulated the same IFN-enhancing trend, further confirming the role of PTEN in promoting IFN-I signaling (Fig. S1B through E).

To assess whether PTEN modulates coronavirus replication, PTEN-knockdown Huh7 cells were infected with HCoV-229E, HCoV-OC43, or SARS-CoV-2. In all cases, PTEN deficiency significantly enhanced viral replication, as evidenced by increased viral RNA and protein levels (Fig. 1E, F, I, J, M, and N; Fig. S2A through C). Notably, SARS-CoV-2 infection decreased PTEN protein abundance, whereas no such reduction was observed following HCoV-229E or HCoV-OC43 infection (Fig. 1F, J, and N). In addition, we further confirmed the increase in virus in PTEN-knockdown cells by immunofluorescence staining of the nucleocapsid (N) protein of SARS-CoV-2 (Fig. 1G, K, and O). Despite the enhanced intracellular viral replication, it remained unclear whether these viral components could be efficiently assembled into infectious progeny virions. As expected, viral titration showed a significant increase in the production of infectious progeny virions after PTEN knockdown (Fig. 1H, L, and P), suggesting that the enhanced viral RNA and protein were able to efficiently assemble into mature viral particles.

To further investigate the antiviral potential of PTEN, we overexpressed PTEN in Huh7 cells in a dose-dependent manner, followed by infection with the same panel of HCoVs. As anticipated, elevated PTEN expression led to a concentration-dependent decrease in SARS-CoV-2 RNA and protein abundance, as well as a reduction in progeny viral titers (Fig. 1Q through S; Fig. S2D through J). However, overexpression of phosphatase-deficient PTEN (PTEN-C124S) no longer significantly inhibited virus replication, indicating that the antiviral ability of PTEN depends on its phosphatase activity (Fig. 1Q through S). These data strongly support the conclusion that PTEN suppresses the replication of multiple HCoVs, likely by enhancing IFN-I responses and impairing efficient production of infectious virions (Fig. 1T).

### SARS-CoV-2 promotes degradation of PTEN by ubiquitin-proteasome pathway

**Fig 2:**
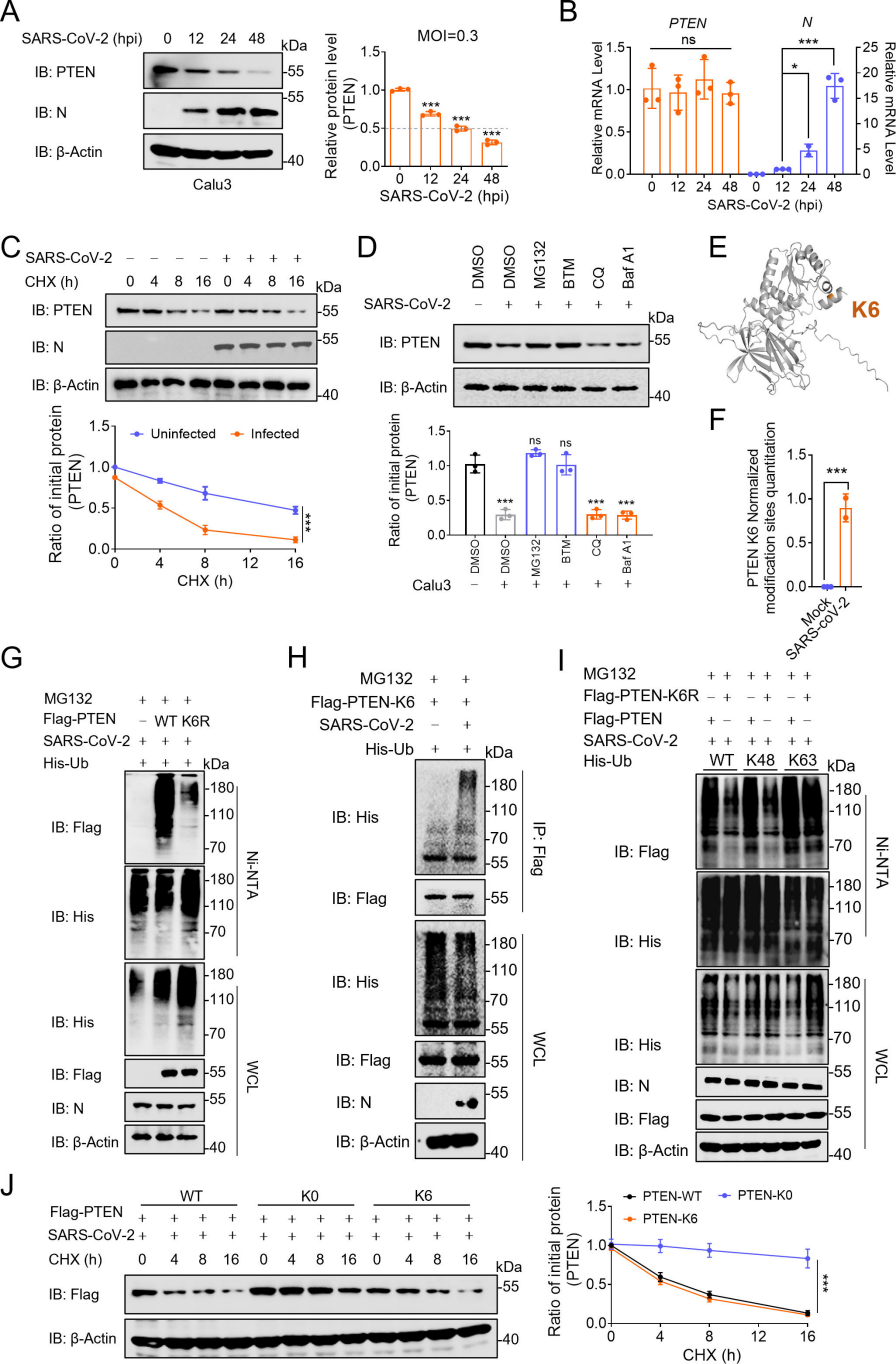
SARS-CoV-2 infection mediates the ubiquitinated degradation of PTEN. (**A, B**) Calu3 cells infected with SARS-CoV-2 were collected at 12, 24, and 48 h to measure PTEN protein by Western blot (**A**) and mRNA by RT-qPCR (**B**). (**C**) Calu3 cells were infected with SARS-CoV-2, and non-infected Calu3 cells were used as a control. Cells were treated with CHX (25 μg/mL), 12 h after infection, and cell protein lysates were collected after 0, 4, 8, and 16 h for PTEN detection. (**D**) HEK293T cells were treated with dimethyl sulfoxide (DMSO), CHX (25 μg/mL), MG132 (20 μM), bortezomib (BTM) (10 μM), chloroquine (CQ) (20 μM), and Baf A1 (1 μM) for 8 h and then collected. Protein levels of PTEN were detected by Western blotting. (**E**) Illustration of K6 location in PTEN. (**F**) Calu3 cells were subjected to mock infection and SARS-CoV-2 (MOI = 1) infection, and cell lysates were collected 24 h later for mass spectrometry analysis. Mass spectrometry identified ubiquitination at PTEN K6. (**G**) Flag-PTEN and Flag-PTEN-K6R were co-expressed with His-Ub in HEK293T-hACE2 cells, which were infected with SARS-CoV-2 (MOI = 0.1) for 24 h and treated with MG132 (20 μM) for 8 h before collection. The ubiquitin chain of PTEN was analyzed by Ni-NTA pull-down and Western blot. (**H**) HEK293T-hACE2 cells expressing Flag-PTEN-K6 and His-Ub were infected with SARS-CoV-2 (MOI = 0.1) for 24 h and then treated with MG132 (20 μM) for 8 h. The ubiquitin chain of PTEN was analyzed by Flag immunoprecipitation and Western blotting. (**I**) HEK293T-hACE2 cells co-transfected with PTEN and ubiquitin mutants (Lys48 or Lys63) were infected with SARS-CoV-2 (MOI = 0.1) for 24 h and then treated with MG132 (20 μM) for 8 h. The ubiquitin chain of PTEN was analyzed by Ni-NTA pull-down and Western blot. (**J**) In HEK293T-hACE2 cells, Flag-PTEN, PTEN-K0, and Flag-PTEN-K6 were expressed and infected with SARS-CoV-2. After 12 h, the cells were treated with CHX, and the expression of Flag-PTEN-K6 was detected by Western blot. Protein lysates of the cells were collected at 4, 8, and 16 h and tested for PTEN. Data are expressed as mean ± SD from a minimum of three independent assays. Statistical significance was determined using unpaired Student’s *t*-test, with ns indicating *P* > 0.05, *P < 0.05, **P < 0.01, and ***P < 0.001.

PTEN’s stability and activity are closely controlled by the ubiquitin-proteasome system (UPS) (45, 46). In this study, we detected that in Calu3 cells (a human lung epithelial cell line) following SARS-CoV-2 infection, PTEN protein levels showed a significant time- and dose-dependent decrease following SARS-CoV-2 infection (Fig. 2A; Fig. S3A), while the expression of PTEN mRNA remained relatively stable (Fig. 2B). Furthermore, in order to confirm that the degradation rate of PTEN increased after SARS-CoV-2 infection, we treated the cells with cycloheximide (CHX) to block the synthesis of new proteins, so as to more directly evaluate the stability and degradation kinetics of PTEN protein during infection. The results showed that the degradation rate of PTEN protein in the SARS-CoV-2 infection group was significantly higher than that in the non-infection group (Fig. 2C).

To investigate the specific pathway of PTEN degradation, we used MG132 and bortezomib (BTM) to block the proteasomal pathway or chloroquine (CQ) and bafilomycin A1 (Baf A1) to block the lysosomal pathway in HEK293T cells. Notably, only proteasome inhibition restored PTEN stability, confirming that PTEN degradation is mediated by the proteasome rather than the lysosome (Fig. 2D; Fig. S3B and C).

Proteins degraded by the proteasome pathway are usually mediated by ubiquitination. To determine the specific ubiquitination sites of PTEN, we analyzed the previous ubiquitin proteomics data. These data were obtained by enriching whole-cell lysates with anti-ubiquitin antibody and conducting mass spectrometry detection after Calu3 cells were infected with SARS-CoV-2. These data detail the ubiquitination sites of each protein and the number of identified peptides (47). We marked the ubiquitination sites of PTEN in the structural schematic diagram (Fig. 2E). Meanwhile, the number of ubiquitinated peptides corresponding to PTEN K6 was reanalyzed before and after infection. The results showed that PTEN K6 was ubiquitinated upon SARS-CoV-2 infection. However, this ubiquitination was not detected under uninfected conditions, indicating that K6 ubiquitination is specifically induced by viral infection (Fig. 2F).

To elucidate the level and major type of ubiquitination at PTEN K6, several PTEN mutants were constructed, including Flag-PTEN-K6R (lysine 6 mutated to arginine); Flag-PTEN-K0 (all lysine sites mutated to arginine); Flag-PTEN-K6 (only the sixth lysine is retained). *In vivo* ubiquitination assays using these mutants showed that mutation of the K6 site markedly reduced PTEN ubiquitination, even in the presence of SARS-CoV-2 (Fig. 2G). Conversely, when K6 was retained as the only lysine residue in PTEN, SARS-CoV-2 was still able to induce robust ubiquitination of PTEN (Fig. 2H). In addition, we also constructed mutant ubiquitin plasmids (His-Ub-K48: Lys48 only; His-Ub-K63: Lys63 only), and found that the PTEN K6 site mainly underwent K48-type ubiquitination modification, which is a typical signal for proteasomal degradation (Fig. 2I).

Therefore, we examined the effect of the PTEN K6 site on its protein stability. The results showed that the mutation of PTEN K6 (Flag-PTEN-K6R) significantly prolonged the half-life of PTEN (Fig. S3D), and the MG132 treatment could no longer significantly restore the protein level of PTEN (Fig. S3E). These results indicate that ubiquitination at K6 reduces PTEN stability during SARS-CoV-2 infection. To further verify the functional relevance of the K6 locus, Flag-PTEN, Flag-PTEN-K0, and Flag-PTEN-K6 were expressed in HEK293T-hACE2 cells and infected with SARS-CoV-2. The results showed that SARS-CoV-2 infection resulted in a significant reduction in the protein stability of Flag-PTEN and Flag-PTEN-K6 but not Flag-PTEN-K0, indicating that K6 is necessary and sufficient for SARS-CoV-2-induced PTEN degradation (Fig. 2J).

### STUB1 is a key E3 ligase mediating PTEN K6 ubiquitination

**Fig 3:**
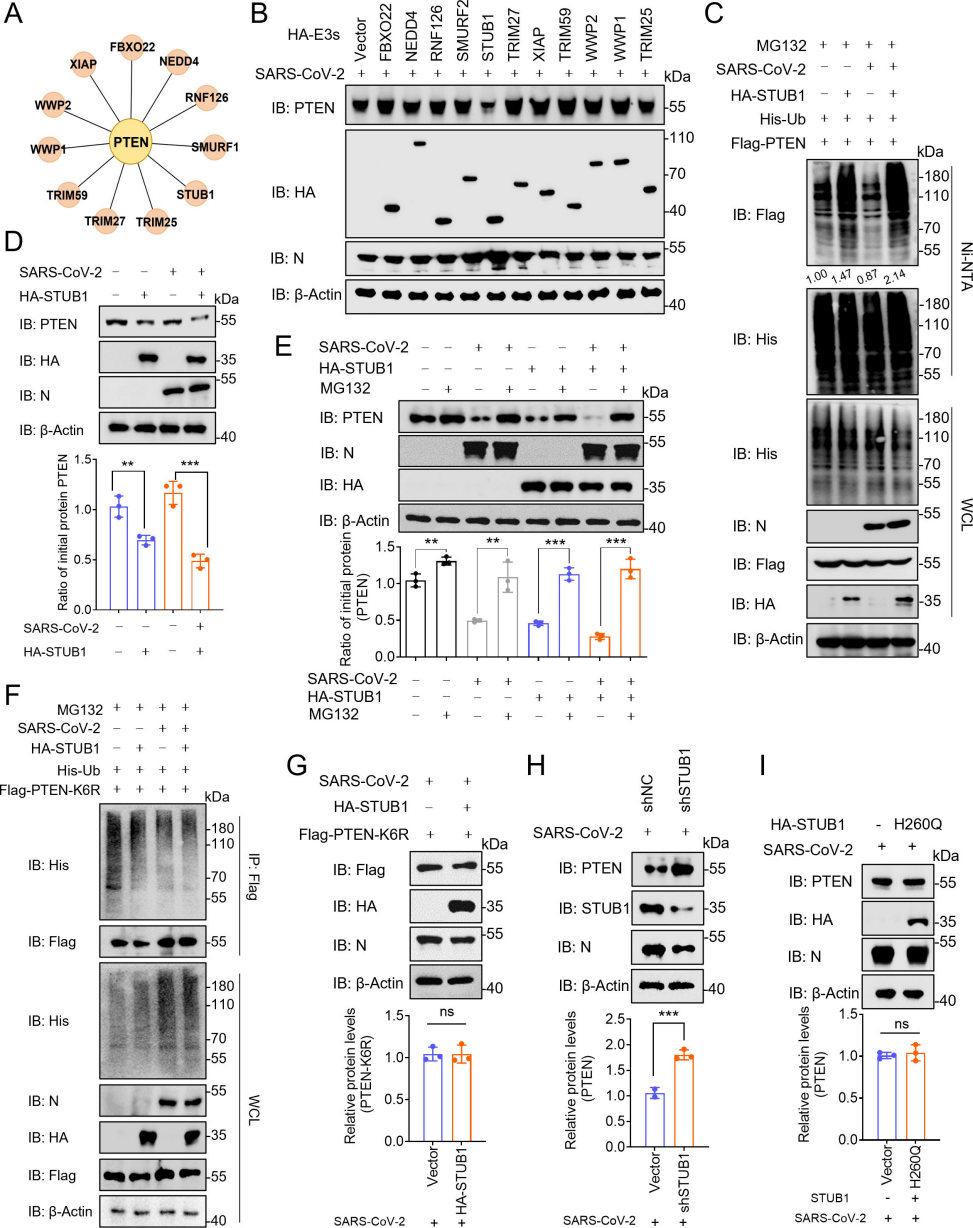
The E3 ligase STUB1 mediates PTEN ubiquitination at K6. (**A**) Predicted E3 ligases targeting PTEN based on Ubibrowser analysis. (**B**) Individual expression of these E3 ligases in HEK293T-hACE2 cells followed by SARS-CoV-2 (MOI = 0.1) infection; PTEN levels were assessed 24 h post-infection. (**C**) HEK293T-hACE2 cells expressing Flag-PTEN and His-Ub were transfected with vector or HA-STUB1, infected with SARS-CoV-2 (MOI = 0.1) for 24 h, and treated with MG132 (20 μM) for an additional 8 h. PTEN ubiquitination was examined by Flag immunoprecipitation and Western blotting, followed by gray-scale analysis quantification of PTEN ubiquitination signals. (**D**) Calu3 cells transfected with empty vector or HA-STUB1 were infected with SARS-CoV-2 (MOI = 1) or mock; PTEN expression was measured 24 h later. (**E**) After plasmid transfection and infection (MOI = 1), Calu3 cells were treated with DMSO or MG132 (20 μM) before protein extraction to analyze PTEN and STUB1 by Western blotting. (**F**) HEK293T-hACE2 cells expressing Flag-PTEN-K6R and His-Ub were transfected with vector or HA-STUB1, infected with mock or SARS-CoV-2 (MOI = 0.1) for 24 h, and then treated with MG132 (20 μM) for 8 h. Flag immunoprecipitation and Western blot analysis of the ubiquitin chain of PTEN. (**G**) Expression of Flag-PTEN-K6R and HA-STUB1 in SARS-CoV-2-infected (MOI = 1) Calu3 cells was assessed at 24 h. (**H**) STUB1 was knocked down using shRNA and infected with SARS-CoV-2 (MOI = 1), and cell lysates were collected for protein levels as shown. (**I**) Expression of HA-STUB1-H260Q in SARS-CoV-2 infected (MOI = 1) Calu3 cells was assessed at 24 h, and cell lysates were collected for protein levels as shown. Data are expressed as mean ± SD from a minimum of three independent assays. Statistical significance was determined using an unpaired Student’s *t*-test, with ns indicating *P* > 0.05, *P < 0.05, **P < 0.01, and ***P < 0.001.

To clarify the molecular mechanism of PTEN ubiquitination and degradation, we constructed all the reported PTEN-related E3 ligase-expressing plasmids with HA tag (Fig. 3A) (48–55). Transfection of these E3 ligase-expressing plasmids into HEK293T-hACE2 cells and SARS-CoV-2 infection showed that only STUB1 significantly reduced the PTEN protein levels (Fig. 3B; Fig. S4A and B), which indicates that STUB1 may function as a key E3 ligase mediating PTEN degradation during SARS-CoV-2 infection.

To validate this hypothesis, we confirmed a physical interaction between STUB1 and PTEN, consistent with earlier reports (Fig. S4C) (56). Moreover, STUB1 significantly enhanced PTEN ubiquitination, particularly under SARS-CoV-2 infection (Fig. 3C). Correspondingly, STUB1 overexpression led to a robust reduction in PTEN abundance, which was exacerbated upon SARS-CoV-2 infection and reversed by the proteasome inhibitor MG132 (Fig. 3D and E), indicating a proteasome-dependent mechanism.

To determine whether STUB1 mediates ubiquitination of the PTEN K6 residue, we coexpressed STUB1 with a PTEN mutant with K6 replaced by arginine (PTEN-K6R). This mutation markedly attenuated STUB1-mediated PTEN ubiquitination and preserved protein stability (Fig. 3F and G; Fig. S4D). Furthermore, knockdown of STUB1 using either shRNA or siRNA in Calu3 cells significantly reduced PTEN ubiquitination and elevated its protein levels (Fig. 3H; Fig. S5A through F), supporting STUB1’s role as a key regulator of PTEN stability. To assess whether this regulation is dependent on STUB1’s enzymatic activity, we generated a catalytically inactive mutant (STUB1-H260Q), which failed to induce PTEN degradation (Fig. 3I). Finally, dual-luciferase reporter assays demonstrated that wild-type STUB1 (STUB1-WT), but not the inactive H260Q mutant (STUB1-H260Q), potently suppressed IFN-I production (Fig. S5G and H). This suggests that STUB1 inhibition of PTEN-mediated enhancement of IFN-I responses is dependent on its ubiquitinase activity, further linking STUB1 to antiviral immune modulation.

Together, these findings identify STUB1 as the principal E3 ligase that mediates PTEN ubiquitination at K6 during SARS-CoV-2 infection. This ubiquitin-dependent degradation of PTEN contributes to viral immune evasion by dampening IFN-I responses.

### STUB1 promotes SARS-CoV-2 replication in an E3 ligase activity-dependent manner

**Fig 4:**
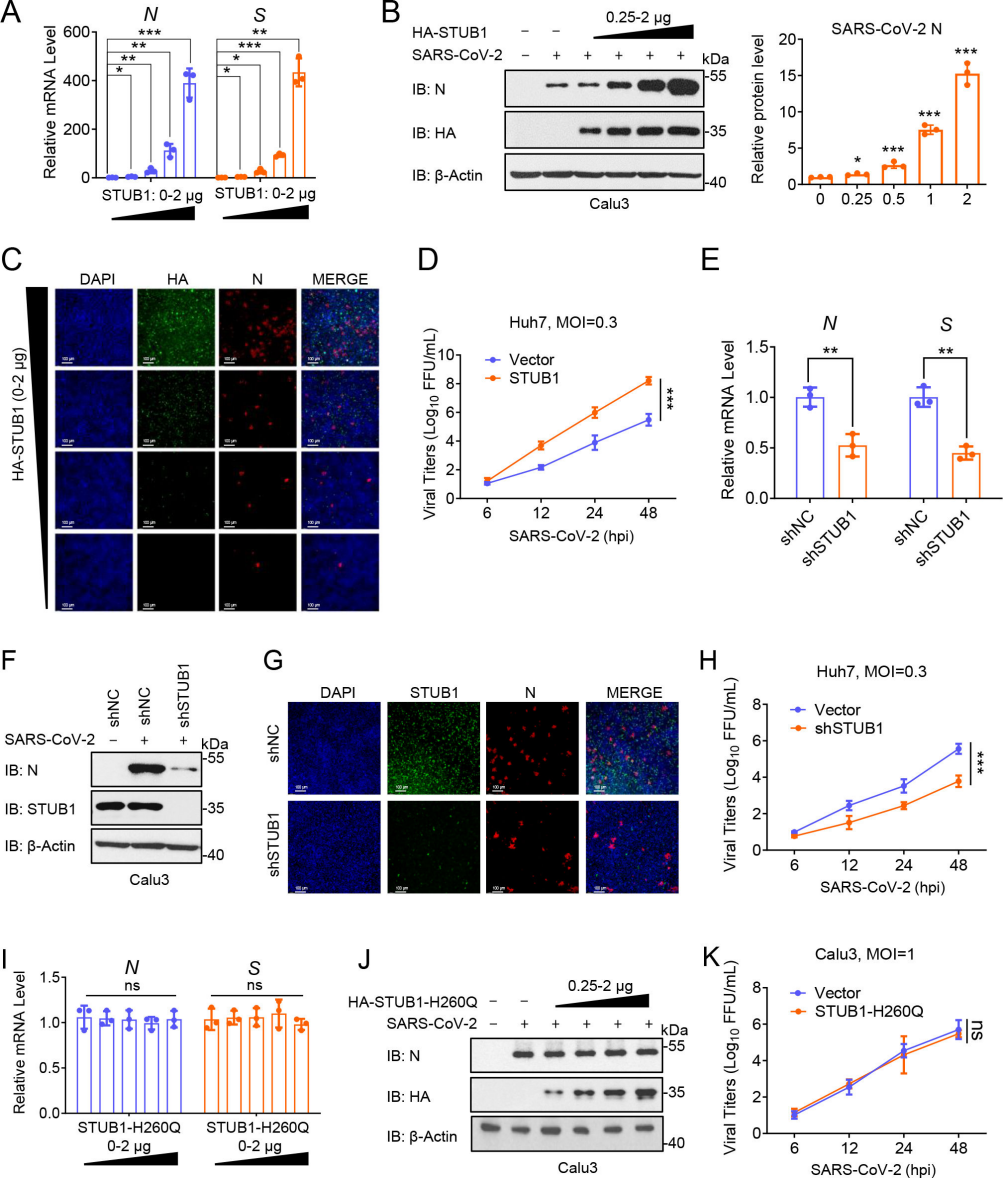
STUB1 enhances SARS-CoV-2 replication. (**A**) Calu3 cells were transfected with increasing amounts of STUB1 plasmid (0, 0.25, 0.5, 1, and 2 μg) prior to SARS-CoV-2 infection (MOI = 1). Viral *N* and *S* gene transcripts were quantified by RT-qPCR. (**B**) Parallel experiments assessed viral N protein levels by Western blot after SARS-CoV-2 (MOI = 0.1) infection. (**C**) Calu3 cells transfected with different doses of HA-STUB1 (green) were SARS-CoV-2 (MOI = 1, 24 h) infected, and viral replication was assessed by N (red) protein detection, with DAPI indicating nuclei (blue). (**D**) Supernatants from STUB1-overexpressing, SARS-CoV-2 (MOI = 1) infected cells were serially diluted to infect Vero E6 cells; infection progression was monitored at 6, 12, 24, and 48 h by crystal violet staining. (**E**) Calu3 cells with control or STUB1-targeting shRNA were SARS-CoV-2 (MOI = 1) infected, and viral N and S mRNA levels were measured by RT-qPCR after 24 h. (**F**) Calu3 cells transfected with shNC or shSTUB1 were infected with SARS-CoV-2 (MOI = 1, 24 h), and the viral N protein was detected by Western blot. (**G**) Calu3 cells transfected with different doses of shNC or shSTUB1 (green) were SARS-CoV-2 (MOI = 1, 24 h) infected, and viral replication was assessed by N (red) protein detection, with DAPI indicating nuclei (blue). (**H**) Supernatants from shSTUB1, SARS-CoV-2 (MOI = 0.3) infected cells were serially diluted to infect Vero E6 cells; infection progression was monitored at 6, 12, 24, and 48 h by crystal violet staining. (**I, J**) Calu3 cells transfected with increasing amounts of catalytically inactive STUB1-H260Q mutant plasmid were SARS-CoV-2 (MOI = 1, 24 h) infected; viral RNA (**I**) and protein (**J**) levels were measured by RT-qPCR and Western blot, respectively. (**K**) Infectious viral titers from STUB1-H260Q Calu3 supernatants were assessed by infecting Vero E6 with serial dilutions, followed by staining at indicated times. Data are expressed as mean ± SD from a minimum of three independent assays. Statistical significance was determined using an unpaired Student’s *t*-test, with ns indicating *P* > 0.05, *P < 0.05, **P < 0.01, and ***P < 0.001.

After confirming that STUB1 inhibits IFN-I production by promoting PTEN degradation, we proceeded to investigate how STUB1 influences SARS-CoV-2 replication. We performed graded overexpression of STUB1 in Calu3 and HeLa-hACE2 cells. A dose-dependent increase in viral replication was observed, as indicated by the increased RNA and protein levels of SARS-CoV-2 *N* (Fig. 4A and B; Fig. S6A through C). These findings were corroborated by immunofluorescence staining, which revealed increased viral N expression in STUB1-overexpressing cells (Fig. 4C; Fig. S6D). Furthermore, supernatants from STUB1-overexpressing cells contained significantly higher levels of infectious progeny virus, as measured by subsequent infection of Vero E6 cells (Fig. 4D; Fig. S6E).

To further confirm the proviral role of STUB1, we employed shRNA-mediated knockdown of STUB1 in Calu3 cells, followed by SARS-CoV-2 infection. Knockdown of STUB1 resulted in a substantial reduction in SARS-CoV-2 replication, as confirmed by measurements of viral RNA, protein levels, and infectious titers (Fig. 4E through H; Fig. S6F through H). Given that STUB1 mediates PTEN degradation via its E3 ubiquitin ligase activity, we next examined whether this enzymatic function is required for enhancing viral replication. Expression of a catalytically inactive STUB1 mutant (H260Q) failed to promote SARS-CoV-2 replication, in contrast to wild-type STUB1 (Fig. 4I through K). To demonstrate that the promotion of SARS-CoV-2 replication by STUB1 depends on the degradation of PTEN, we conducted verification in cells knocked down with PTEN and found that the replication of SARS-CoV-2 was no longer affected by the expression level of STUB1 (Fig. S6I and J).

These results suggest that STUB1 promotes PTEN degradation through the ubiquitin-proteasome pathway in an E3 ligase activity-dependent manner, thereby inhibiting IFN-I production and the associated antiviral response and ultimately promoting SARS-CoV-2 replication.

### ORF3a enhances STUB1-mediated ubiquitination and degradation of PTEN

**Fig 5:**
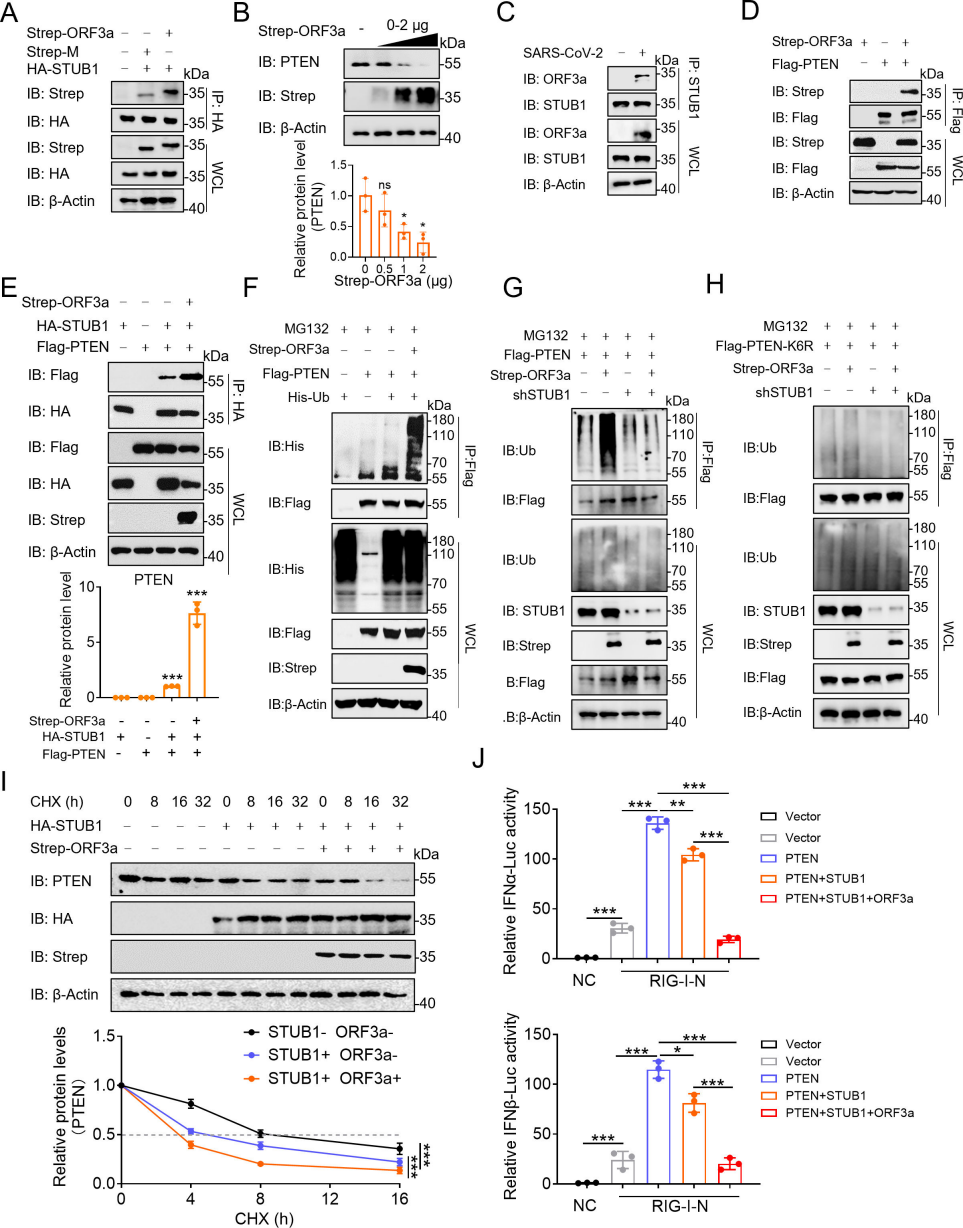
ORF3a promotes STUB1 ubiquitination for PTEN degradation. (**A**) HEK293T cells transfected with indicated plasmids were lysed, and immunoprecipitation with anti-HA beads was performed; Strep-tagged M and ORF3a proteins were detected by Western blot. (**B**) Dose-dependent effect of ORF3a on PTEN protein levels was analyzed by Western blot in HEK293T cells transfected with increasing amounts of ORF3a plasmid. (**C**) Calu3 cells were either mock-infected or infected with SARS-CoV-2 (MOI = 1, 24 h), and then the cell lysates were collected and treated with anti-STUB1 antibody and Protein G overnight at 4°C shaking table. The final proteins that were pulled down were used to detect SARS-CoV-2 ORF3a using Western blot. (**D**) Immunoprecipitation of Strep-ORF3a using anti-Flag beads was performed in transfected cells, followed by Western blot detection. (**E**) HA immunoprecipitation evaluated the interaction between Strep-ORF3a and Flag-PTEN. (**F–H**) HEK293T-hACE2 cells expressing the plasmid depicted in the figure were infected with mock or SARS-CoV-2 (MOI = 0.1) for 24 h, and then treated with MG132 (20 μM) for 8 h. Flag immunoprecipitation and Western blot analysis of the ubiquitin chain of PTEN. (**I**) Western blot analyzed PTEN protein levels post-transfection with indicated plasmids. (**J**) Calu3 cells expressing IFNα or IFNβ luciferase reporters were co-transfected with PTEN alone, PTEN plus STUB1, or PTEN, STUB1, and ORF3a; following RIG-I-N stimulation, luciferase activity was measured to assess promoter activation. Data are expressed as mean ± SD from a minimum of three independent assays. Statistical significance was determined using an unpaired Student’s *t*-test, with ns indicating *P* > 0.05, *P < 0.05, **P < 0.01, and ***P < 0.001.

Given that SARS-CoV-2 infection promotes PTEN ubiquitination and STUB1 is the only one tested that ubiquitinates PTEN, we speculate that specific viral proteins are involved in triggering this process. To identify potential viral cofactors, we constructed 20 stably expressed SARS-CoV-2 proteins and co-expressed them with STUB1 to evaluate their interactions. Among these, both the membrane (M) protein and ORF3a exhibited detectable interactions with STUB1 (Fig. 5A; Fig. S7A through F). Notably, only ORF3a expression, but not that of the M protein, resulted in a pronounced decrease in PTEN protein levels, suggesting that ORF3a is a key viral factor that promotes STUB1-mediated PTEN ubiquitination at K6 (Fig. 5B; Fig. S7G and H).

Furthermore, to test whether ORF3a interacts with STUB1 under SARS-CoV-2 infection, Calu3 cells were either mock-infected or infected with SARS-CoV-2 and examined by an anti-STUB1 pull-down assay. The results confirm the interaction between STUB1 and ORF3a under natural infection conditions (Fig. 5C). Since STUB1 interacts with PTEN and ORF3a, respectively, we next investigated whether ORF3a directly binds to PTEN. Co-immunoprecipitation confirmed the interaction between ORF3a and PTEN (Fig. 5D; Fig. S7I). Moreover, the presence of ORF3a significantly enhanced the interaction between STUB1 and PTEN (Fig. 5E; Fig. S7J). In addition, ORF3a also significantly enhanced the ubiquitination level of PTEN (Fig. 5F). To clarify whether ORF3a-mediated PTEN ubiquitination is STUB1-dependent, we performed validation in STUB1-knockdown cells, where ORF3a no longer promoted PTEN ubiquitination when STUB1 was knocked down by shRNA (Fig. 5G). Furthermore, ORF3a no longer significantly enhanced the overall ubiquitination of PTEN when tested using PTEN-K6R, with or without STUB1 knockdown (Fig. 5H). This suggests that ORF3a uses STUB1 to significantly enhance the level of ubiquitination at PTEN K6.

To test the effect of ORF3a on PTEN stability, we performed CHX chase assays. ORF3a expression significantly shortened the half-life of PTEN, indicating accelerated degradation (Fig. 5I). To determine whether ORF3a suppresses the enhanced IFN-I response mediated by PTEN, we examined IFN-I (IFNα and IFNβ) in the presence or absence of ORF3a. The results indicated that ORF3a further inhibited the IFN-I response (Fig. 5J). We reviewed a large number of existing reports and found no reports on the ubiquitination of PTEN K6, suggesting that this might be a new ubiquitination site. To identify the possible reasons for the modification of this site, we used AlphaFold3 to model the structures of PTEN-STUB1 and PTEN-STUB1-ORF3a. By comparing the two models, it was found that ORF3a induced a rearrangement of the N-terminal conformation of PTEN, exposing the K6 residue, which might be the cause of ubiquitination at this site (Fig. S7K). We also provided a structural model with STUB1 and ORF3a concealed to more clearly display the conformational changes at the N-terminal of PTEN (Fig. S7L). However, whether ubiquitination at PTEN K6 is caused by ORF3a-induced conformational changes remains to be experimentally validated.

Collectively, these findings reveal that SARS-CoV-2-encoded ORF3a promotes STUB1-mediated ubiquitination and proteasomal degradation of PTEN, thereby dampening host interferon responses and facilitating viral replication.

### Oroxin B inhibits SARS-CoV-2 replication

Previous studies have reported that Oroxin B can increase PTEN protein level and inhibit PI3K/AKT signaling axis by downregulating miR-221 to relieve its translation inhibition on PTEN 3′UTR-mediated by miR-221 (57, 58). SF1670 was used as an inhibitor of PTEN by inhibiting its enzymatic activity and regulating PIP3 signaling and elevated AKT phosphorylation (59). Although PTEN-targeting compounds have primarily been developed as anticancer agents, their antiviral potential remains largely unexplored. We evaluated the impact of Oroxin B and SF1670 on SARS-CoV-2 replication in cells. Cytotoxicity assays confirmed that both compounds, at concentrations below 10 μM, did not affect cell viability (Fig. S8A and B). Following SARS-CoV-2 infection, Oroxin B-treated cells exhibited significantly reduced levels of viral N gene RNA and protein, and progeny virus titers compared to DMSO-treated controls, whereas SF1670 treatment enhanced viral replication (Fig. S8C through E). These findings suggest that pharmacological modulation of PTEN activity influences the efficiency of SARS-CoV-2 replication.

**Fig 6:**
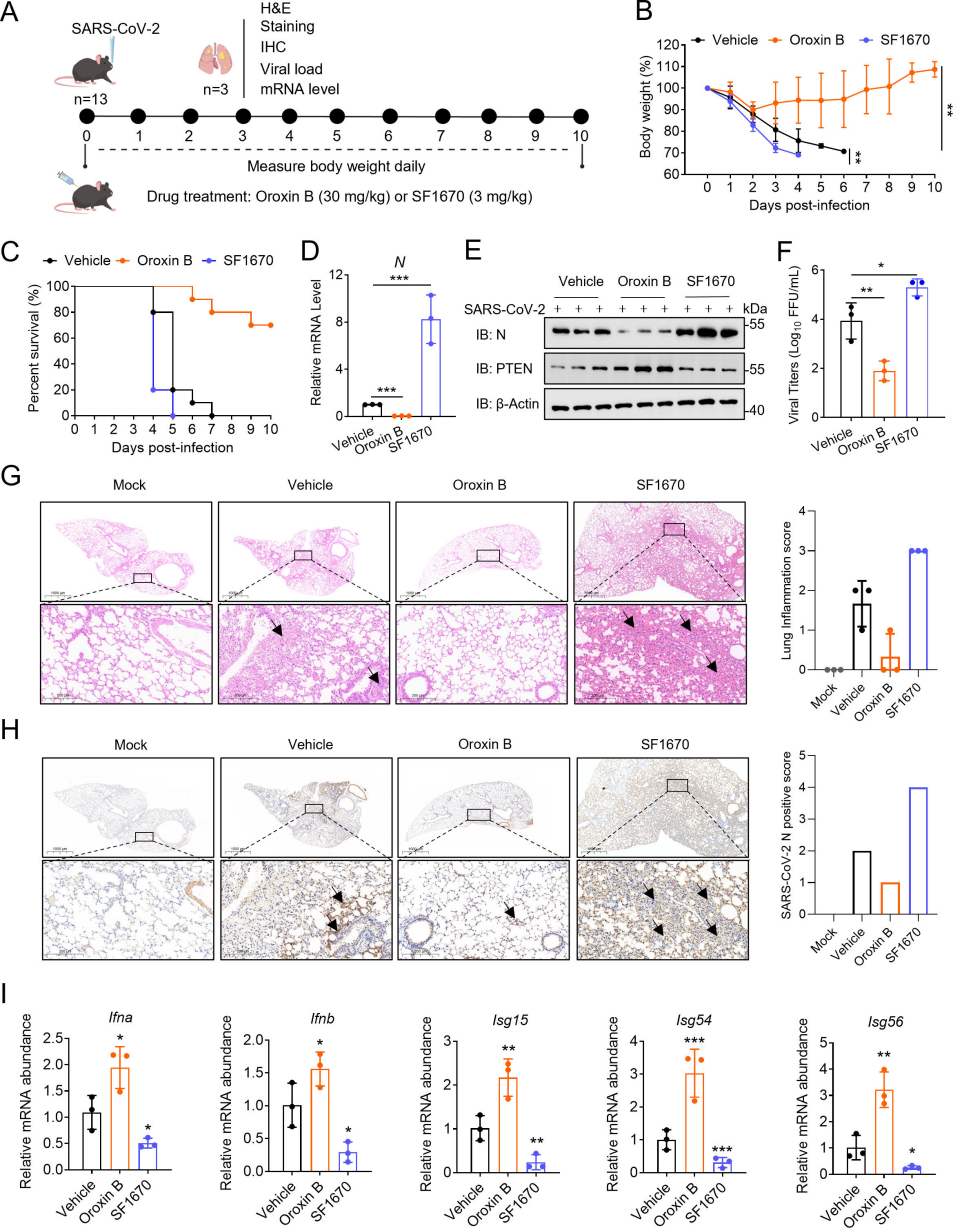
Agonists of PTEN inhibit SARS-CoV-2 replication. (**A**) K18-hACE2 mice were intranasally challenged with 1 × 10^4^ FFU of wild-type SARS-CoV-2 and received once-daily intraperitoneal injections of vehicle control, Oroxin B (30 mg/kg), or SF1670 (3 mg/kg) (*n* = 13). On the 3rd day of infection, three mice from each group were randomly selected to collect lung tissues for detection. (**B**) Body weight changes were monitored throughout infection and treatment (*n* = 10), and the curves of mouse body weight changes were re-subjected to two-way ANOVA. (**C**) Changes in mouse survival were monitored throughout infection and treatment (*n* = 10). (**D, E**) Oroxin B or SF1670 was administered simultaneously with SARS-CoV-2 infection, and lung RNA and protein samples were harvested at 72 h post-infection to measure viral *N* gene expression. (**F**) Lung homogenates from treated and infected mice were serially diluted and used to infect Vero E6 cells for 48 h; cells were fixed with 4% paraformaldehyde and stained with 0.1% crystal violet containing 1% paraformaldehyde to evaluate viral titers. (**G**) H&E staining of lung tissue sections from different treatment groups. (**H**) IHC staining of lung tissues using an antibody targeting SARS-CoV-2 N protein across experimental groups. According to the immunohistochemical results, the proportion of N protein-positive cells was scored as 0: <5%, 1: 5%–25%, 2: 25%–50%, 3: 50%–75%, and 4: 75%–100%. (**I**) Quantification of *Ifna*, *Ifnb*, *Isg15*, *Isg54,* and *Isg56* mRNA levels in lung tissues from treated mice. Data are expressed as mean ± SD from a minimum of three independent assays. Statistical significance was determined using unpaired Student’s *t*-test, with *P < 0.05, **P < 0.01, and ***P < 0.001.

To further evaluate the *in vivo* therapeutic potential of PTEN activation, K18-hACE2 transgenic mice were intranasally challenged with SARS-CoV-2 (1 × 10^4^ PFU) and subsequently administered Oroxin B (30 mg/kg) or SF1670 (3 mg/kg) via intraperitoneal injection once daily for 10 consecutive days. Viral burden and lung pathology were assessed at 3 dpi, and the changes in body weight and mortality were tracked continuously for 10 days after infection (Fig. 6A). Oroxin B-treated mice lost less body weight than vehicle- or SF1670-treated mice and began to recover on the 3rd day after infection. However, the vehicle and SF1670 groups showed sustained weight loss, which was accompanied by a higher mortality rate (Fig. 6B and C). In lung tissues, Oroxin B treatment resulted in lower RNA and protein levels of SARS-CoV-2 N. Equal amounts of lung homogenates were used for secondary infection of Vero E6 cells, and Oroxin B-treated mice had lower viral loads (Fig. 6D through F). Histopathological analysis (hematoxylin and eosin [H&E] staining) showed that the Oroxin B treatment group had reduced inflammatory injury in lung tissue, while the SF1670 treatment group had aggravated inflammatory injury in lung tissue (Fig. 6G). Immunohistochemical (IHC) staining showed that the expression of SARS-CoV-2 N in lung tissues of Oroxin B-treated mice was significantly lower than that of vehicle-treated mice (Fig. 6H). Furthermore, higher expression of IFNα, IFNβ, ISG15, ISG54, and ISG56 was detected in the lung tissue of the Oroxin B group (Fig. 6I). Moreover, we found that Oroxin B-treated mice had lower expression of inflammatory genes, which could effectively reduce the tissue damage caused by viral infection (Fig. S8F through I).

In conclusion, these results indicate that the pharmacological upregulation of PTEN by Oroxin B inhibits the replication of SARS-CoV-2 and alleviates virus-induced lung injury, highlighting its potential as a novel antiviral treatment strategy. However, SF1670 inhibited the enzymatic activity of PTEN, thus leading to more severe SARS-CoV-2 infection.

## DISCUSSION

PTEN plays critical roles in regulating cellular metabolism, proliferation, and immune responses (60). Li et al. reported a critical role for PTEN in the induction of IFN-I, a hallmark of antiviral innate immunity, independent of the kinase PI(3)K and AKT pathways. PTEN controls the negative phosphorylation site of IRF3 at Ser97, and the release of this negative regulation by PTEN phosphatase activity is essential for IRF3 activation and IRF3 translocation into the nucleus. PTEN controls the translocation of IRF3 into the nucleus, a major transcription factor responsible for IFNβ production (25). Despite its well-characterized functions in oncogenesis, the role of PTEN during HCoV infection has remained largely unexplored. In our study, we provide evidence that PTEN exerts antiviral activity against multiple HCoVs, highlighting its potential as a key but underappreciated element of the innate immune system. Mechanistically, these antiviral effects were accompanied by enhanced IFN-I production, suggesting that PTEN acts as a critical upstream regulator of antiviral innate immunity during coronavirus infection.

Considering that SARS-CoV-2 utilizes diverse mechanisms to counteract host antiviral defenses, we next investigated whether it interferes with PTEN stability. Our results revealed a marked decrease in PTEN protein levels following SARS-CoV-2 infection, without corresponding changes in its mRNA expression, implicating a post-translational mechanism. Through a targeted screen of known PTEN-associated E3 ubiquitin ligases, we identified STUB1 as the only ligase that robustly mediated PTEN ubiquitination and degradation in infected cells. However, whether additional, as-yet-unidentified E3 ubiquitin ligases also regulate PTEN ubiquitination under infection conditions remains unclear. The E3 ligase activity of STUB1 depends on H260 (61), and we confirmed that PTEN degradation is dependent on both K6 and STUB1 E3 ligase activity by functional analysis of catalytic inactivation of STUB1 mutant (H260Q) and K6 deletion of PTEN mutant (PTEN-K6R). During SARS-CoV-2 infection, these effects became markedly amplified, indicating that the virus exploits the host ubiquitin-proteasome pathway to degrade PTEN, consequently dampening IFN-driven antiviral responses. However, considering that the E3 ubiquitin ligase does not have an absolute one-to-one targeting relationship with the substrate protein, it is possible that STUB1 promotes viral replication through additional mechanisms, which need to be verified by subsequent studies.

To dissect the viral factors involved in this process, we screened 20 SARS-CoV-2 proteins and identified ORF3a as a critical modulator. ORF3a can weaken host immune defense and promote the replication and pathogenicity of SARS-CoV-2 at multiple levels by interfering with membrane transport, selectively inhibiting STING-mediated autophagy antiviral response, reducing MHC-I surface expression, and activating inflammatory and cell death pathways. ORF3a was found to directly interact with both STUB1 and PTEN, enhancing their association and promoting PTEN ubiquitination. Structural modeling further suggested that ORF3a may induce a conformational shift in the N-terminal region of PTEN, facilitating exposure of the K6 residue and increasing its susceptibility to STUB1-mediated ubiquitination. Thus, ORF3a acts as a viral effector linking STUB1 and PTEN to promote immune escape by inhibiting IFN-I responses, consistent with previous reports; our study further elucidates the underlying mechanism (62). Among the seven known HCoVs, only SARS-CoV and SARS-CoV-2 encode the ORF3a protein and share about 72% amino acid sequence similarity. Therefore, the mechanism identified in this study, in which ORF3a promotes PTEN degradation via STUB1-mediated ubiquitination, may not be conserved among other HCoVs. However, this study did not construct SARS-CoV-2 virus strains with ORF3a deletion. Validation using an ORF3a-deletion virus would substantially strengthen the conclusions of this study. In addition, we have not determined whether SARS-CoV ORF3a has a similar function, and it remains possible that other coronaviruses may induce PTEN ubiquitination and degradation through distinct mechanisms.

To explore the therapeutic relevance of targeting this pathway, we employed Oroxin B, a known PTEN activator (58, 63), and SF1670 (64, 65), a PTEN inhibitor. Oroxin B treatment significantly augmented IFN-I responses and suppressed SARS-CoV-2 replication, while SF1670 had the opposite effect. Importantly, in K18-hACE2 transgenic mice, intraperitoneal administration of Oroxin B not only alleviated weight loss and pulmonary pathology but also increased PTEN protein levels and reduced viral titers in the lungs. These observations were further supported by immunohistochemical analyses showing reduced viral protein expression in Oroxin B-treated animals. Together, these data suggest that pharmacological stabilization of PTEN could serve as a viable therapeutic approach to combat SARS-CoV-2 and potentially other coronaviruses.

In summary, our study identifies a novel mechanism by which SARS-CoV-2 evades host innate immunity via STUB1-mediated degradation of PTEN, a process potentiated by the viral accessory protein ORF3a. These findings highlight the critical role of PTEN as an antiviral restriction factor and provide mechanistic insight into how a key host regulator is selectively targeted by a viral immune antagonist. By elucidating the ORF3a-STUB1-PTEN axis, we expand the current understanding of virus-host interactions and offer a new conceptual framework for therapeutic intervention aimed at preserving or restoring host antiviral defenses.

Nonetheless, our study has certain limitations. While we focused primarily on PTEN stability and its role in IFN-I signaling, it remains to be determined whether PTEN also exerts antiviral effects through other pathways, such as regulation of autophagy, cell death, or metabolic reprogramming. In addition, the predicted conformational changes induced by ORF3a in PTEN’s N-terminal region, particularly the exposure of the K6 ubiquitination site, require validation using high-resolution structural techniques such as cryo-electron microscopy or NMR spectroscopy. Finally, future studies should further define the role of PTEN in different immune cell subsets and determine whether it interacts with additional viral proteins to more comprehensively delineate its contribution to host antiviral defense.

### Conclusion

**Fig 7:**
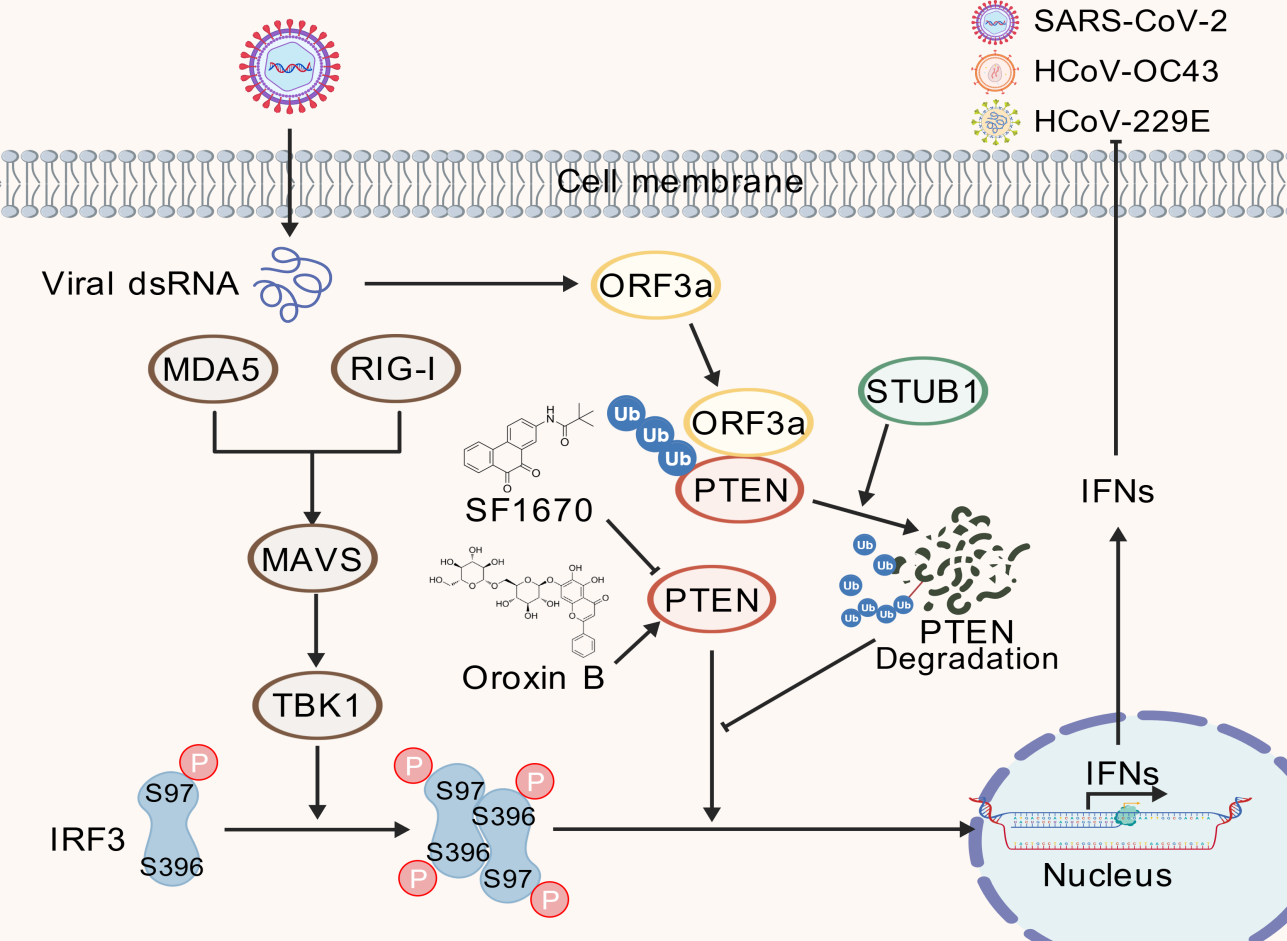
Schematic diagram of the mechanism by which SARS-CoV-2 ORF3a inhibits host antiviral immunity by promoting STUB1-mediated ubiquitination and degradation of PTEN by altering the conformation of PTEN protein.

This study demonstrates that PTEN acts as an antiviral factor against multiple HCoVs by enhancing innate immune responses. However, SARS-CoV-2 subverts this antiviral defense by promoting ubiquitination and degradation of PTEN at lysine 6 via the E3 ligase STUB1, a process further facilitated by the viral ORF3a protein. Importantly, activation of PTEN by Oroxin B effectively boosts antiviral immunity *in vivo*, highlighting PTEN as a promising therapeutic target. These findings provide valuable mechanistic insights into coronavirus immune evasion strategies and open new avenues for the development of antiviral interventions (Fig. 7).

## MATERIALS AND METHODS

### Cell culture

Huh7, a cell line that can be infected by a variety of human coronaviruses, is a useful model for studying mechanisms common to a variety of coronaviruses. Calu3 (human lung epithelial cell line) is a high-quality cell line for SARS-CoV-2 research. HeLa-hACE2 and HEK293T-hACE2 cells stably express human ACE2 receptor in the original HeLa or HEK293T cell lines, which have the advantage of easy transfection. They are the cell lines constructed in our laboratory for SARS-CoV-2 research. All cells were cultured at 37°C in the presence of 5% CO₂ in Dulbecco’s Modified Eagle Medium (Gibco, 11965092), supplemented with 10% fetal bovine serum (BioIndustries, 04-001-1) and 100 μg/mL penicillin-streptomycin. HeLa-hACE2 and HEK293T-hACE2 cells were generated in-house by transducing HeLa and HEK293T cells with the human ACE2 receptor gene.

### Mice

Female K18-hACE2 mice (6 to 8 weeks of age, Cyagen Biosciences, S240325) received 1 × 10^4^ PFU of SARS-CoV-2 by nasal instillation on day 0. For *in vivo* administration, Oroxin B and SF1670 were freshly prepared in vehicle consisting of 10% DMSO, 40% PEG300, 5% Tween-80, and 45% saline. Oroxin B was formulated at 3 mg/mL for a dosing regimen of 30 mg/kg, and SF1670 was formulated at 0.3 mg/mL for a dosing regimen of 3 mg/kg. Both compounds were administered by intraperitoneal injection at a volume of 10 mL/kg once daily. On day 3 post-infection, three mice from each group were randomly selected to collect lung tissues for detection. Body weight was recorded daily, and mice were euthanized when they lost more than 25% of their body weight until day 10 post-infection, when all mice were euthanized.

### Immunoprecipitation and His pull-down

Cells were washed with phosphate-buffered saline (PBS) and lysed on ice for 1 h in a denaturing buffer containing 100 mM NaH₂PO₄, 10 mM Tris-HCl (pH 8.0), 8 M urea, 500 mM NaCl, 0.1% Triton X-100, 10% glycerol, 10 mM β-mercaptoethanol (β-ME), a protease inhibitor cocktail, and 10 mM imidazole. The clarified lysates were incubated with anti-Flag, anti-HA, anti-Strep beads, or Ni-NTA agarose resin (Invitrogen, OTI4C5, 88836, 10103D, 600442) for 3 h at room temperature with gentle rotation. Beads were washed using the same lysis buffer to eliminate non-specific binding. Bound proteins were released with an elution buffer containing 30% glycerol, 5% SDS, 0.72 M β-ME, 0.15 M Tris-HCl (pH 6.7), and 200 mM imidazole. The eluates were separated by SDS-PAGE and subjected to Western blot analysis.

### Antibodies and plasmids

Antibodies: anti-PTEN (Abcam, ab260011), anti-HA (TransGen Biotech, HT301-01), anti-His (TransGen Biotech, F1804), and anti-Strep tag II (Abcam, ab76949). Antibodies targeting viral proteins included anti-N (SARS-CoV-2) (Sino Biological, 11675-T62), anti-N (HCoV-OC43) (Abclonal, A20610), anti-N (HCoV-229E) (Sino Biological, 40640-T62), anti-ORF3a (SARS-CoV-2) (Cell Signaling Technology, #34340), and anti-M (SARS-CoV-2) (Cell Signaling Technology, #15333). Additionally, anti-Flag (TransGen Biotech, HT201-01), anti-STUB1 (Sino Biological, 12496-R034), anti-β-actin (TransGen Biotech, HC201), and HRP-conjugated secondary antibodies anti-mouse IgG (TransGen Biotech, HS201-01) and anti-rabbit IgG (TransGen Biotech, HS101-01) were employed for Western blotting and immunodetection.

Plasmids: Plasmids of FBXO22, NEDD4, RNF126, SMURF2, STUB1, TRIM27, XIAP, TRIM59, WWP2, WWP1, and TRIM25 were purchased from SunV Biotechnology Co., Ltd. in Shenzhen, China. Subsequently, the gene fragments were cloned into pcDNA3.1 vectors with HA tags. Plasmids encoding Strep-ORF3a, Flag-tagged PTEN, M, and Strep-N proteins were obtained from Sino Biological (Beijing, China). The Flag-PTEN-K6R mutant was generated by site-directed mutation based on the wild-type Flag-PTEN construct. His-tagged ubiquitin (His-Ub) and its mutant variants were funded and constructed by this laboratory. The viral protein plasmid sequence was referred to the SARS-CoV-2 complete genome sequence provided by NC_045512, and linked to the pCDNA3.1 vector backbone through *Bam*HI and *Xho*I restriction sites, and a Strep tag was added to the C-terminus of the protein. Short hairpin RNAs targeting PTEN (sh-PTEN: CCACAAATGAAGGGATATAAA) and STUB1 (shRNA1: GAAGAGGAAGAAGC

GAGACAT; shRNA2: GCCAATCTGCAGCGAGCTTAC; shRNA3: GCAGTCTGT

GAAGGCGCACTT), as well as siRNAs against STUB1 (siRNA-1: GGAAGACUAUGAAGAAUAATT; siRNA-2: GCGUGUACCUUGAAGAAUA; siRNA-3: GGAUUUGAGUGAUGAUGAATT), were designed and constructed in our laboratory.

### HCoVs

All experiments involving HCoV-229E and HCoV-OC43 were conducted in biosafety level 2 laboratories, whereas SARS-CoV-2-related work was performed in biosafety level 3 facilities. The complete genome sequence of the SARS-CoV-2 strain SZTH-003, originally isolated from a COVID-19 patient, is available in the Global Initiative on Sharing Avian Influenza Data (accession no. EPI_ISL_406594, https://db.cngb.org/gisaid/). HCoV-229E and HCoV-OC43 were obtained from the American Type Culture Collection (VR-740, VR-1558). After a single amplification in Vero E6 cells, viral stocks were aliquoted and stored at −80°C for later use. For infection experiments, HeLa-hACE2, Huh7, HEK293T-hACE2, Calu3, and Vero E6 cells were inoculated with SARS-CoV-2 at the specified MOI for 1 h. Following adsorption, the inoculum was removed, fresh culture medium was added, and cells were maintained at 37°C with 5% CO₂ until collection at designated time points for downstream analyses.

### Cellular immunofluorescence

Glass slides were placed at the bottom of a 24-well culture plate and seeded with cells at ~30% confluency. After 24 h of attachment and subsequent viral infection, cells were rinsed with PBS (Takara, T900) and fixed in 4% paraformaldehyde for 30 min. Following fixation, samples were washed with PBS and permeabilized in 0.5% Triton X-100 (Sangon Biotech, A110694) in PBS for 15 min. After another PBS wash, cells were blocked with 5% BSA (Beyotime, ST025) for 1 h at room temperature. Primary antibodies were then applied and incubated overnight at 4°C with gentle agitation. The next day, primary antibodies were removed, and the cells were washed three times in PBS (10 min each). Nuclei were counterstained with DAPI (Beyotime, C1102), followed by a final PBS wash. Images were captured using a Leica confocal microscope.

### Real-time quantitative PCR

Total RNA was extracted using TRIzol reagent according to standard protocols and then reverse-transcribed into cDNA using BeyoRT II cDNA first strand synthesis kit (RNase H-) (D7168S, Beyotime). This was followed by PCR using the SYBR Green Quantitative RT-qPCR kit (QR0100, Sigma-Aldrich). All procedures were performed according to the instructions provided by the manufacturer. Primer sequences are in the Table S1.

### Construction and detection of luciferase reporter genes

Promoter activities of IFNα, IFNβ, ISG15, IL-6, and CXCL1 were measured using pGL6-based luciferase reporter constructs. Cells were co-transfected with the indicated firefly luciferase reporter plasmids and the pRL-TK Renilla luciferase plasmid as an internal control. After the indicated treatments, firefly and Renilla luciferase activities were measured using the Beyotime dual-luciferase assay kit, and promoter activity was expressed as the firefly/Renilla ratio.

### Protein half-life determination by cycloheximide chase assay

Cells were seeded in 6 cm culture dishes and grown to ~70% confluence prior to transfection. For each dish, 4 µg of plasmid DNA encoding either wild-type PTEN or the indicated mutant was mixed with polyethylenimine and incubated according to the manufacturer’s recommendations to allow complex formation before being added to the cells. Twelve hours after transfection, cells were detached with trypsin, resuspended in complete medium, and redistributed evenly into 12-well plates. Following a further 24 h incubation, when cultures had reached ~90% confluence, cycloheximide (CHX; 30 µg/mL) was added to block new protein synthesis. For experiments assessing proteasome involvement, MG132 (20 µM) was applied simultaneously with CHX. Cells were harvested at the indicated time points, lysed in RIPA buffer supplemented with protease inhibitors, and processed for Western blot analysis to monitor the degradation kinetics of the target protein.

### H&E staining and inflammation score

Tissue samples were fixed in 4% paraformaldehyde, embedded in paraffin, and sectioned at 4 μm–5 μm thickness. After deparaffinization in xylene and rehydration through a graded ethanol series, the sections were stained with hematoxylin, rinsed in running water, and differentiated if necessary. The sections were then counterstained with eosin, dehydrated through graded ethanol, cleared in xylene, and mounted with neutral resin. Inflammation was scored for H&E staining results according to the following criteria: (i) inflammatory cell infiltration, score 0: almost no inflammatory cells, score 1: mild, scattered, or focal, score 2: moderate, multifocal, obvious aggregation, score 3: severe, large, or diffuse distribution; (ii) destruction of alveolar structure/interstitial thickening, score 0: complete alveolar structure, score 1: mild alveolar septal thickening, score 2: obvious structural disorder, partial alveolar collapse, score 3: extensive collapse or consolidation; (iii) exudation, hemorrhage, and alveolar cavity cells, score 0: none, score 1: mild, score 2: moderate, score 3: severe, massive exudation.

### IHC

Formalin-fixed, paraffin-embedded tissue sections (3 µm–5 µm) were deparaffinized in xylene, rehydrated through graded ethanol, and subjected to heat-induced antigen retrieval in citrate or EDTA buffer. Endogenous peroxidase activity was quenched with 3% hydrogen peroxide, followed by blocking with normal serum. Sections were incubated with the primary antibody at optimized dilutions, then with an HRP-conjugated secondary detection reagent. Signal was visualized using a DAB chromogen, and nuclei were counterstained with hematoxylin. Finally, slides were dehydrated, cleared, mounted, and examined under a light microscope.

### Statistical analysis

All results are expressed as mean ± SD from a minimum of three independent assays. Differences between two groups were assessed using a two-tailed unpaired Student’s *t*-test, while multiple group comparisons were analyzed by two-way ANOVA. Statistical evaluations were performed with GraphPad Prism 7 (GraphPad Software, San Diego, CA, USA), and values of *P* < 0.05 were deemed statistically significant.

## Data Availability

The data sets generated during the course of this study are included in the published article and made available by the corresponding author upon reasonable request.

## SUPPLEMENTAL MATERIAL

Supplemental material jvi.00186-26-s0001.docx Fig. S1 to S8; Table S1.

ASM does not own the copyrights to Supplemental Material that may be linked to, or accessed through, an article. The authors have granted ASM a non-exclusive, world-wide license to publish the Supplemental Material files. Please contact the corresponding author directly for reuse.
