# Supplementary material for: SARS-CoV-2 ORF3a suppresses host antiviral interferon responses by promoting STUB1-mediated PTEN proteasomal degradation
Source: J Virol. 2026 Jun 2;100(6):e00186-26. doi: 10.1128/jvi.00186-26 (PMC13288637; doi:10.1128/jvi.00186-26)
Supplement: Supplemental material — Fig. S1 to S8; Table S1. [file jvi.00186-26-s0001.docx]

**Title page：**

**Type of study: Original Research Article**

**SARS-CoV-2 ORF3a suppresses host antiviral interferon responses by promoting STUB1-mediated PTEN proteasomal degradation**

**Short Running title: ORF3a–STUB1 triggers PTEN loss to blunt immunity**

Lujie Fan^1†^, Xiang Gao^1†^, Wei Feng^1†^, Qiang Huang^1^, Xiafei Wei^1^, Chuwei Yang^1^, Yezi Wu^1^, Xiaotong Shen^1^, Juanjuan Zhao^1^, Yuzheng Zhou^1‡^, Zheng Zhang^1, 2, 3‡^

^1^ Institute for Hepatology, National Clinical Research Center for Infectious Disease, Shenzhen Third People’s Hospital, Department of Biochemistry, the Second Affiliated Hospital, School of Medicine, Southern University of Science and Technology, Shenzhen 518112, Guangdong Province, China.

^2^ Guangdong Key laboratory for anti-infection Drug Quality Evaluation, Shenzhen, Guangdong 518112, China.

^3^ Shenzhen Research Center for Communicable Disease Diagnosis, Treatment of Chinese Academy of Medical Science, Shenzhen, Guangdong 518112, China.

^‡^Correspondence:

Yuzheng Zhou, Email: mzhouyuzheng@163.com;

Zheng Zhang, Email: zhangzheng1975@aliyun.com;

^†^These authors contribute equally.

**Supplementary figures**

**
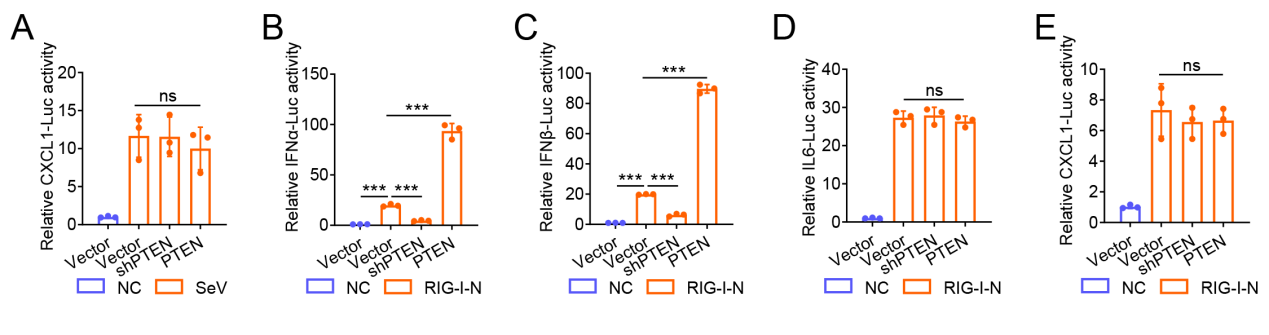
**

**Fig. S1 PTEN upregulated IFN responses, but not inflammatory factors or chemokines.**

(A) In Huh7 cells, luciferase reporter constructs driven by the CXCL1 promoter were individually transfected, along with the pRL-TK vector as an internal control. After 24 hours of transfection, cells were stimulated with Sendai virus (SeV, MOI=1) and incubated for an additional 24 h. Subsequently, cells were lysed, and luciferase activity was measured to assess promoter activation.

(B-E) In Huh7 cells, luciferase reporter constructs driven by the IFN-α, IFN-β, IL-6, and CXCL1 promoter were individually transfected, along with the pRL-TK vector as an internal control. After 24 h of transfection, cells were stimulated with RIG-I-N and incubated for an additional 24 h. Subsequently, cells were lysed, and luciferase activity was measured to assess promoter activation.

Data are expressed as mean ± SD (n = 3 independent experiments). * Indicates *P* < 0.05, ** indicates *P* < 0.01, and *** indicates *P* < 0.001, and statistical significance was determined using *Student's* *t-test*.


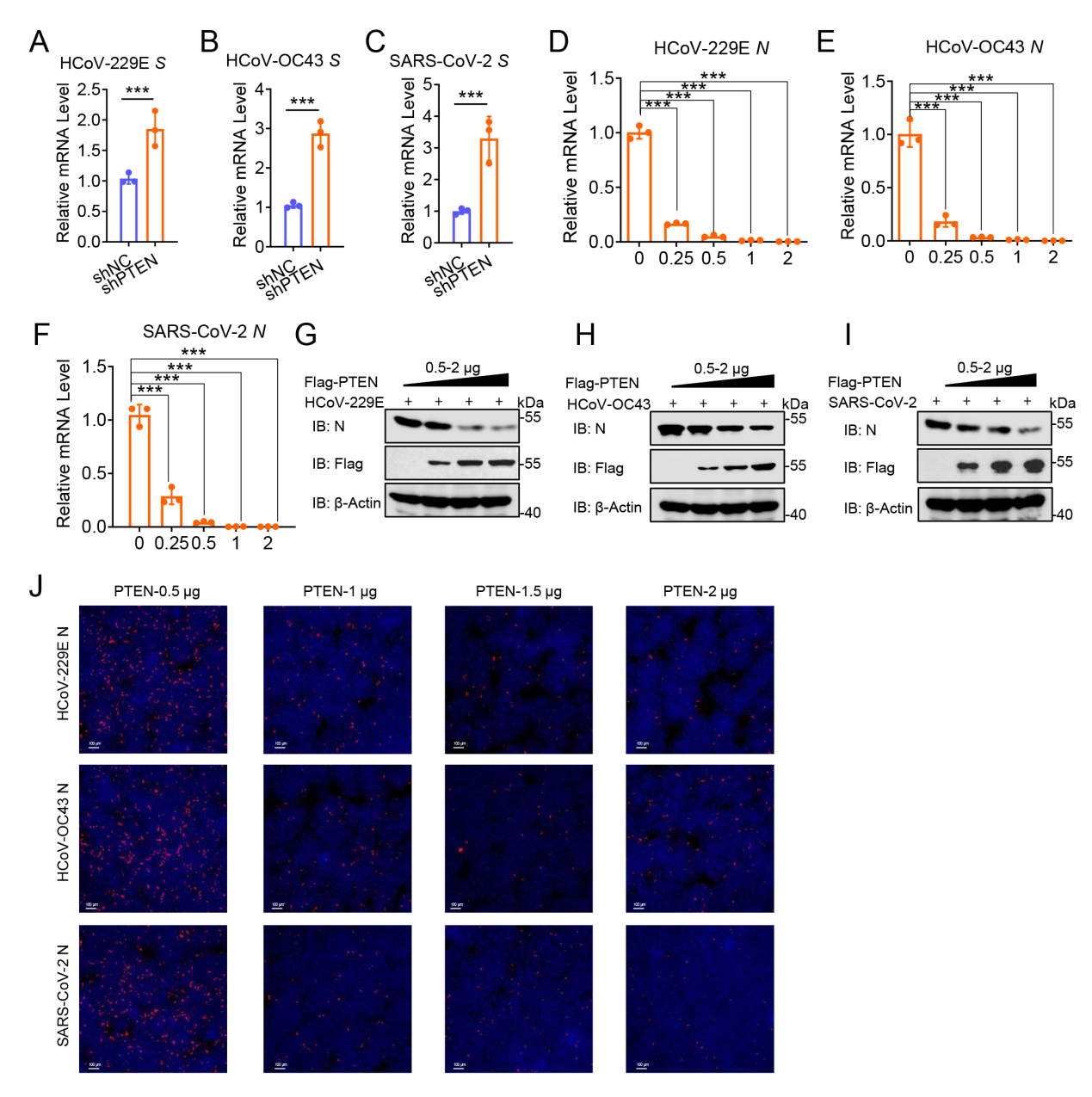


**Fig. S2 PTEN inhibits the replication of a variety of human coronaviruses.**

(A-C) After knocking down PTEN expression in Huh7 cells using shRNA, the cells were infected with HCoV-229E (MOI=0.01, 24 h), HCoV-OC43 (MOI=0.1, 24 h), or SARS-CoV-2 (MOI=0.3, 24 h), respectively. Subsequently, total RNA was extracted to measure viral *Spike (S)* gene mRNA levels.

(D-J) After overexpressing PTEN in Huh7 cells, the cells were infected with HCoV-229E (MOI=0.01, 24 h), HCoV-OC43 (MOI=0.1, 24 h), or SARS-CoV-2 (MOI=0.3, 24 h), respectively. Subsequently, total RNA was extracted to measure viral *Nucleocapsid (N)* gene mRNA levels (D-F), protein lysates were prepared for immunoblotting of the N protein (G-I), and immunofluorescence staining was performed to visualize viral protein expression (J).

Data are expressed as mean ± SD (n = 3 independent experiments). * Indicates *P* < 0.05, ** indicates *P* < 0.01, and *** indicates *P* < 0.001, and statistical significance was determined using *Student's* *t-test*.

**
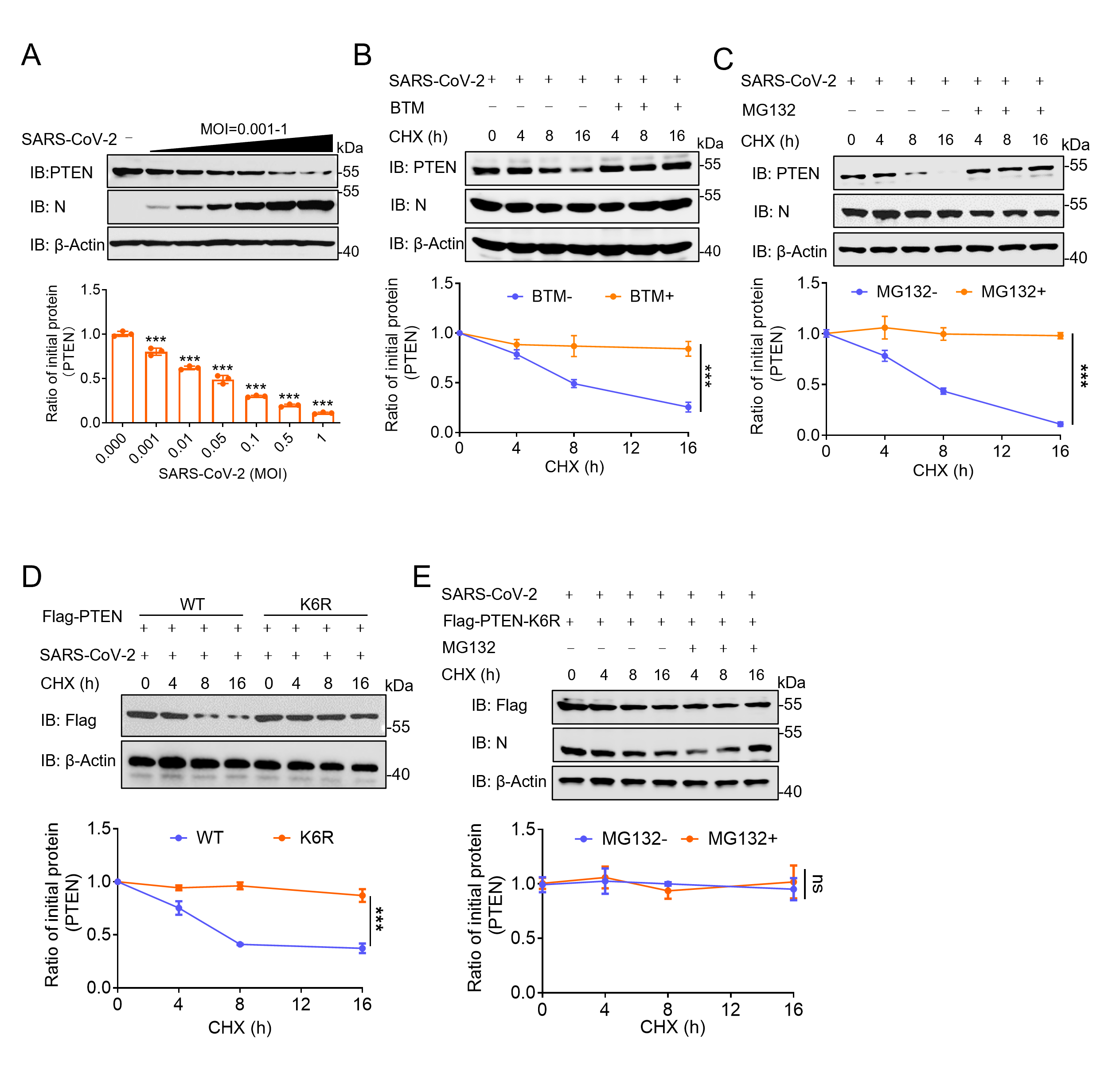
**

**Fig. S3 SARS-CoV-2 mediates the ubiquitin-dependent degradation of PTEN.**

(A) Calu3 cells were infected with SARS-CoV-2 at a MOI of 0.001-1. After 24 h, cell protein lysates were collected and PTEN was detected.

(B) HEK293T-hACE2 cells were transfected with the indicated plasmids and then treated with CHX (25 µg/mL) and BTM (10 µM) and PTEN protein was measured.

(C) HEK293T-hACE2 cells were transfected with the indicated plasmids and then treated with CHX (25 µg/mL) and MG132 (20 µM) and PTEN protein was measured.

(D) HEK293T-hACE2 cells were transfected with the indicated plasmids and then treated with CHX (25 µg/mL) for 0, 4, 8, 16 h and PTEN protein was measured.

(E) HEK293T-hACE2 cells were transfected with the indicated plasmids and then treated with CHX (25 µg/mL) and MG132 (20 µM) for 8 h and PTEN protein was measured.

Data are expressed as mean ± SD (n = 3 independent experiments). * Indicates *P* < 0.05, ** indicates *P* < 0.01, and *** indicates *P* < 0.001, and statistical significance was determined using *Student's* *t-test*.

**
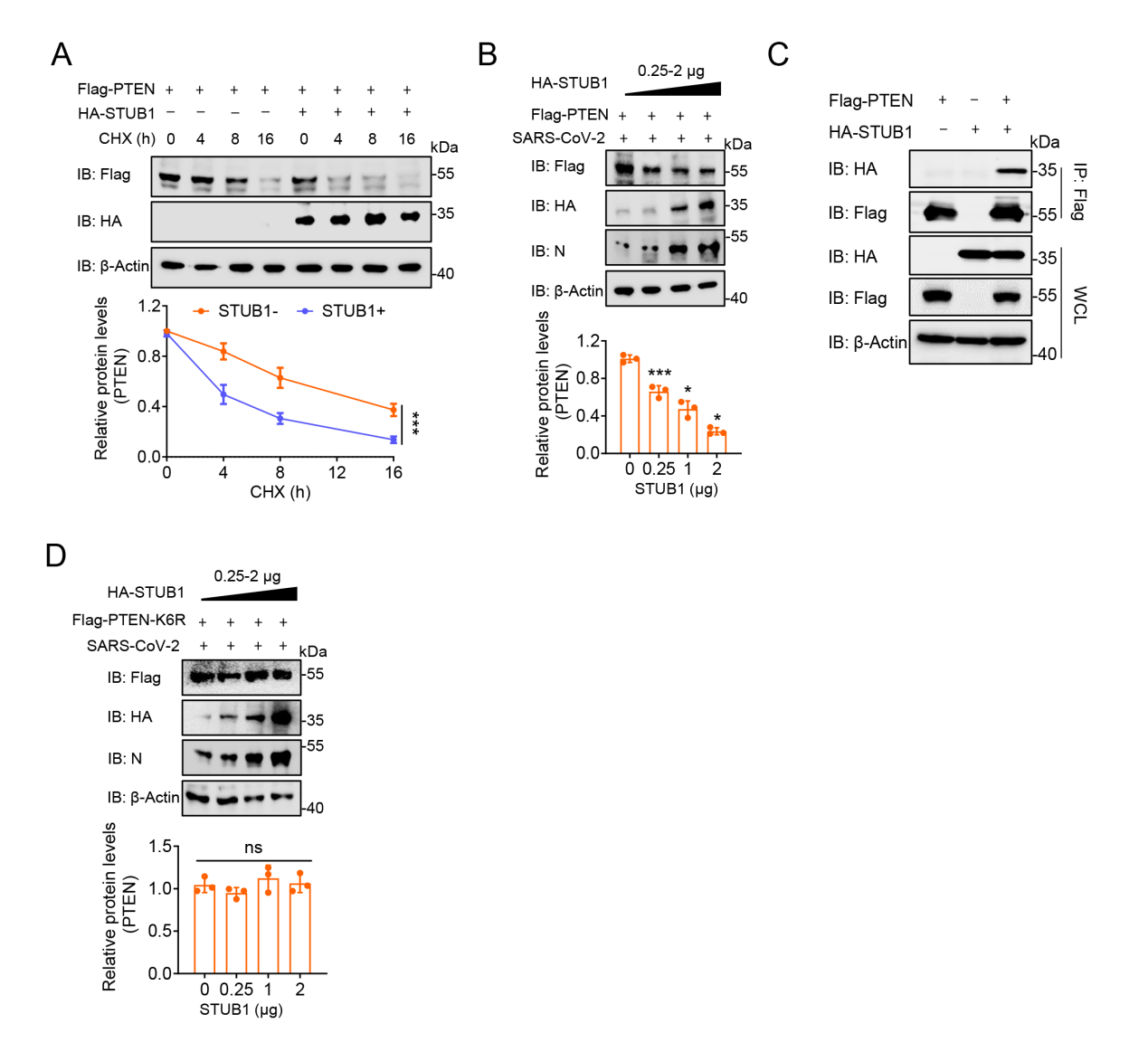
**

**Fig. S4 STUB1 promotes PTEN protein degradation.**

1. HEK293T-hACE2 cells were transfected with vector or STUB1 and then infected with SARS-CoV-2. Cells were treated with CHX (25 µg/mL) 12 h later, and cell protein lysates were collected at 0, 4, 8, and 16 h for PTEN detection.
2. Flag-PTEN was expressed in HEK293T-hACE2 expressing HA-STUB1 and subsequently subjected to SARS-CoV-2 infection, cell protein lysates were collected, and PTEN was detected.
3. Co-immunoprecipitation of Flag-PTEN and HA-STUB1 in HEK293T cells confirmed their interaction.
4. Flag-PTEN-K6R was expressed in HEK293T-hACE2 expressing HA-STUB1 and subsequently subjected to SARS-CoV-2 infection, cell protein lysates were collected, and PTEN was detected.

Data are expressed as mean ± SD (n = 3 independent experiments). * Indicates *P* < 0.05, ** indicates *P* < 0.01, and *** indicates *P* < 0.001, and statistical significance was determined using *Student's* *t-test*.


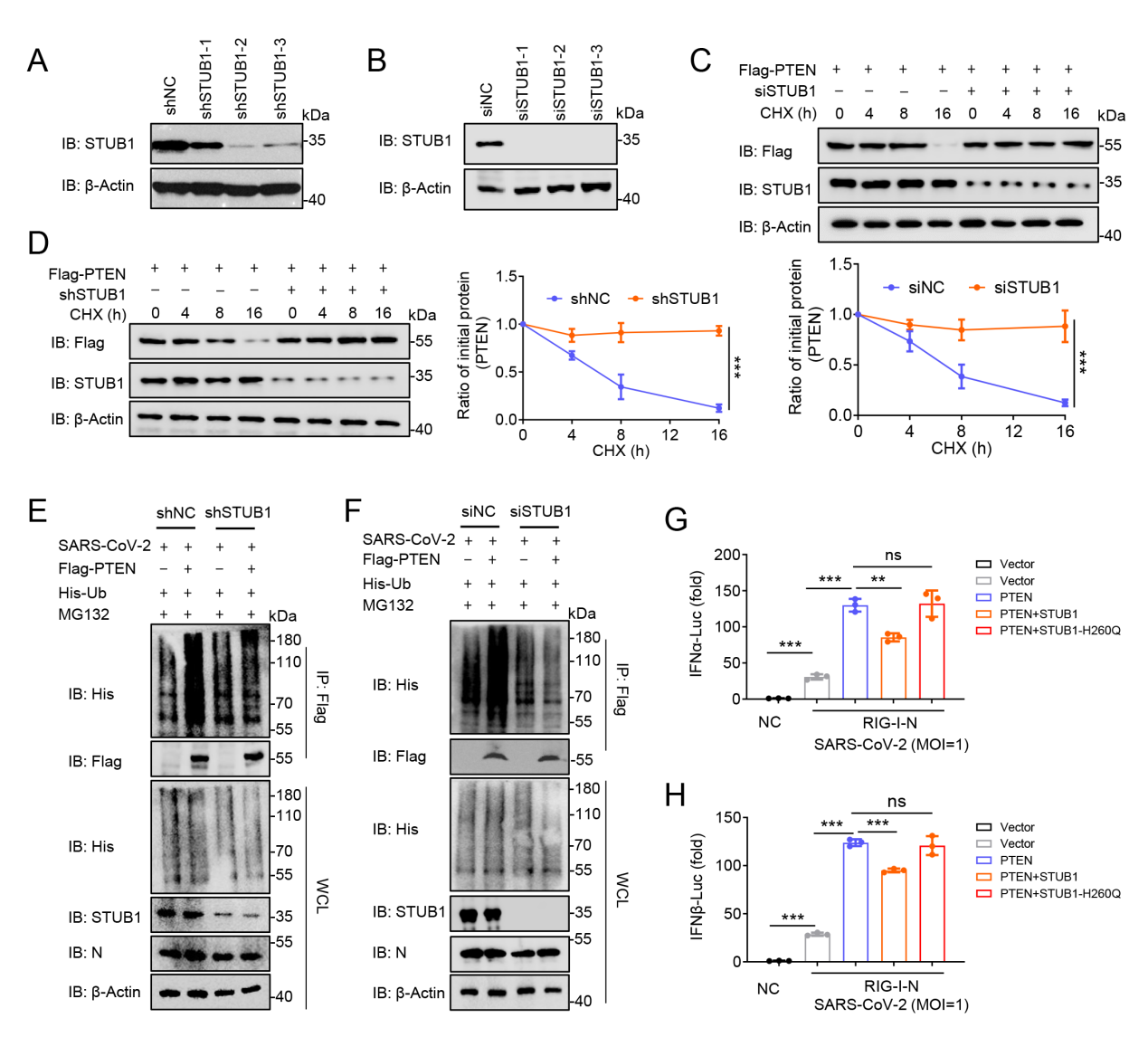


**Fig. S5 Knockdown of STUB1 effectively increased the stability of PTEN protein.**

(A-B) STUB1 was knocked down in HEK293T-hACE2 cells by shRNA or siRNA.

(C-D) After STUB1 knockdown in HEK293T-hACE2 cells using shRNA or siRNA, cells were treated with CHX (25 µg/mL) 12 h later, and protein lysates were collected at 0, 4, 8, and 16 h for PTEN detection.

(E-F) The above plasmids were transfected into HEK293T-hACE2 cells and treated with MG132 (20 µM) for 8 h after 24 h of SARS-CoV-2 (MOI=0.1) infection. Cell lysates were collected, PTEN protein was enriched by Flag antibody, and PTEN ubiquitination was detected by anti-Ub antibody.

(G-H) Calu3 cells expressing luciferase reporter genes driven by IFN-α and IFN-β promoters were transfected with PTEN, PTEN+STUB1 and PTEN+STUB1-H260Q, respectively. The pRL-TK vector was used as an internal control. At 24 h post-transfection, cells were infected with SARS-CoV-2 (MOI=1), stimulated with RIG-I-N, and incubated for an additional 24 h. Subsequently, cells were lysed, and luciferase activity was measured to assess promoter activation.

Data are expressed as mean ± SD (n = 3 independent experiments). * Indicates *P* < 0.05, ** indicates *P* < 0.01, and *** indicates *P* < 0.001, and statistical significance was determined using *Student's* *t-test*.


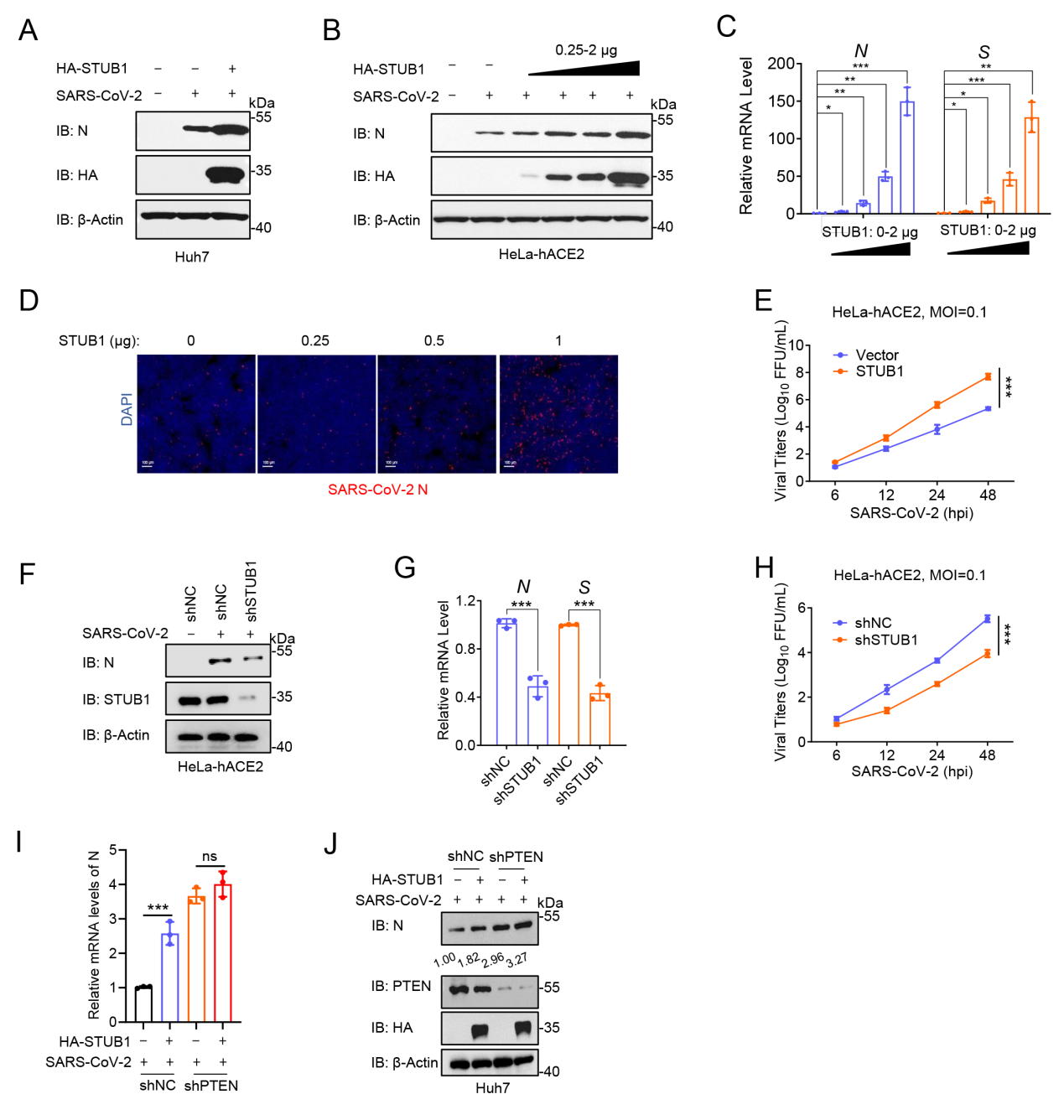


**Fig. S6 STUB1 enhances SARS-CoV-2 replication.**

(A-B) After STUB1 was overexpressed in Huh7 or HeLa-hACE2 cells, the SARS-CoV-2 N protein level was detected after infection with SARS-CoV-2.

1. HeLa-hACE2 cells were transfected with 0, 0.25, 0.5, 1, and 2 µg of STUB1 plasmid, respectively. Then the cells were infected with SARS-CoV-2, and *N* and *S* were detected by RT-qPCR.
2. Immunofluorescence was used to detect SARS-CoV-2 N protein in HeLa-hACE2 cells with gradient overexpression of STUB1.
3. HeLa-hACE2 cells with STUB1 overexpression were infected with SARS-CoV-2 (MOI=0.3) for 24 h. Subsequently, the viral supernatant was used to infect Vero E6 cells for 6, 12, 24, 48 h with serial dilutions, followed by fixation with 4% paraformaldehyde, then staining with 0.1% crystal violet solution containing 1% paraformaldehyde. Error bars represent the SD of technical triplicates.
4. The protein level of SARS-CoV-2 N was detected after STUB1 knockdown by shRNA in HeLa-hACE2.
5. HeLa-hACE2 cells were transfected with shNC and shSTUB1 plasmids, respectively, and then infected with SARS-CoV-2. After 24 h, cellular RNA was collected, and SARS-CoV-2 *N* and *S* were detected by RT-qPCR.
6. HeLa-hACE2 with STUB1 knockdown was infected with SARS-CoV-2 at a MOI=0.3 for 24 h. Vero E6 cells were infected with serial dilutions of the viral supernatant for 6, 12, 24, 48 h, then fixed with 4% paraformaldehyde and stained with 0.1% crystal violet solution containing 1% paraformaldehyde. Error bars indicate the SD of technical triplicates.

(I-J) Vector or HA-STUB1 was expressed in Huh7 cells of shNC and shPTEN, respectively, and then infected with SARS-CoV-2 (MOI=0.3). Cells were collected 24 h later to detect the mRNA and protein levels of SARS-CoV-2 N.

Data are expressed as mean ± SD (n = 3 independent experiments). * Indicates *P* < 0.05, ** indicates *P* < 0.01, and *** indicates *P* < 0.001, and statistical significance was determined using *Student's* *t-test*.

**
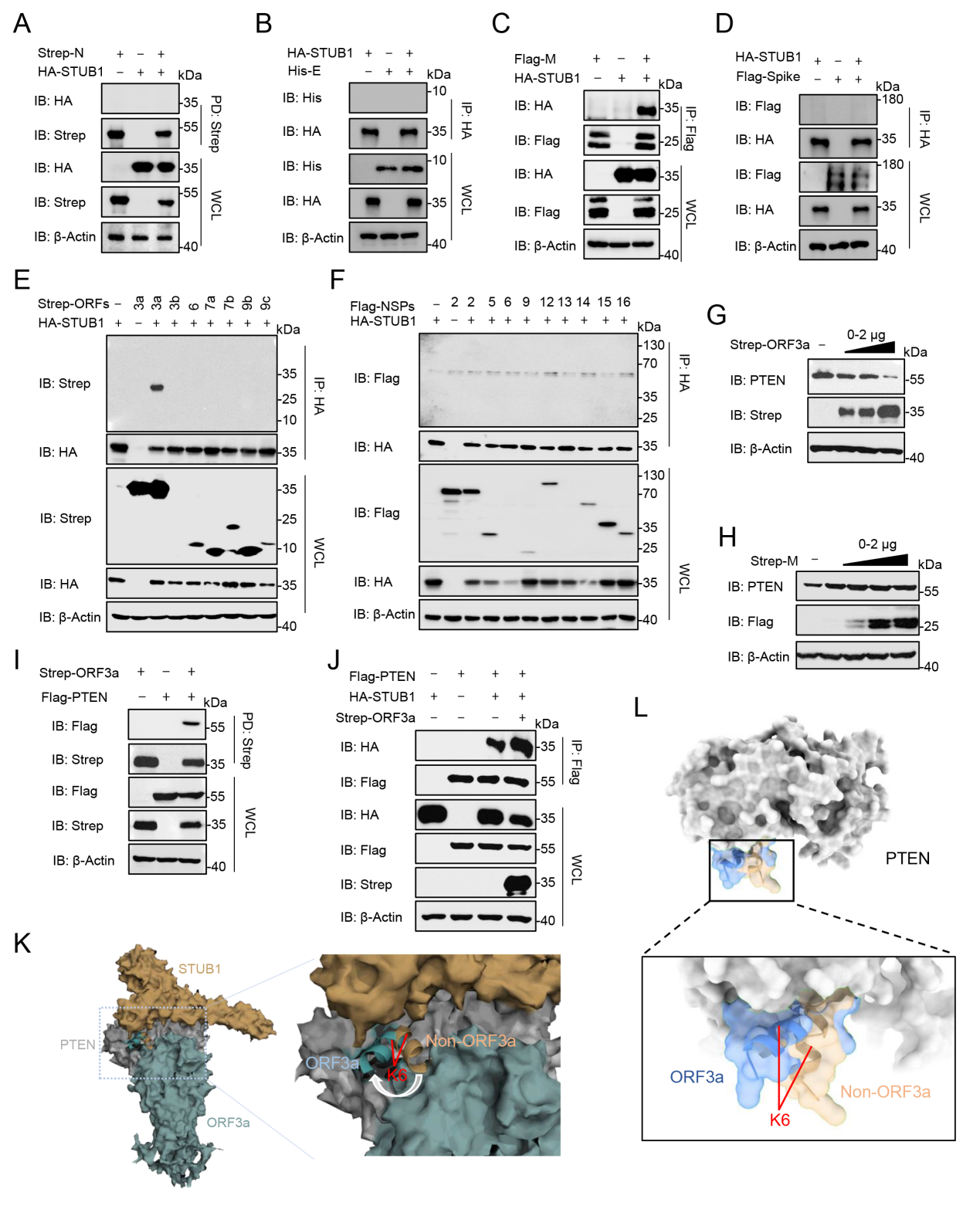
**

**Fig. S7 ORF3a promotes STUB1 ubiquitination for PTEN degradation.**

(A-F) HEK293T cells transfected with plasmids as described were lysed with NP-40. The whole-cell lysates were subjected to pull-down with StrepTactin beads, anti-HA or anti-Flag beads and Western blotting to detect the SARS-CoV-2 proteins.

(G-H) Calu3 cells were transfected with ORF3a at increasing concentrations, and whole-cell protein lysates were collected and tested for PTEN.

(I-J) HEK293T cells transfected with plasmids as described were lysed with NP-40. The whole-cell lysates were subjected to pull-down with anti-Strep or anti-Flag beads and Western blotting to detect Strep-ORF3a and Flag-PTEN.

(K) The structure of the PTEN-STUB1 binary complex predicted by AlphaFold3 and the ternary complex formed by PTEN-STUB1-ORF3a were superimposed to display the complete structural model of the PTEN-STUB1-ORF3a ternary complex to assess PTEN conformational changes.

(L) The structure of the PTEN-STUB1 binary complex predicted by AlphaFold3 and the ternary complex formed by PTEN-STUB1-ORF3a were superimposed, showing only the PTEN structural model to assess conformational changes.

**
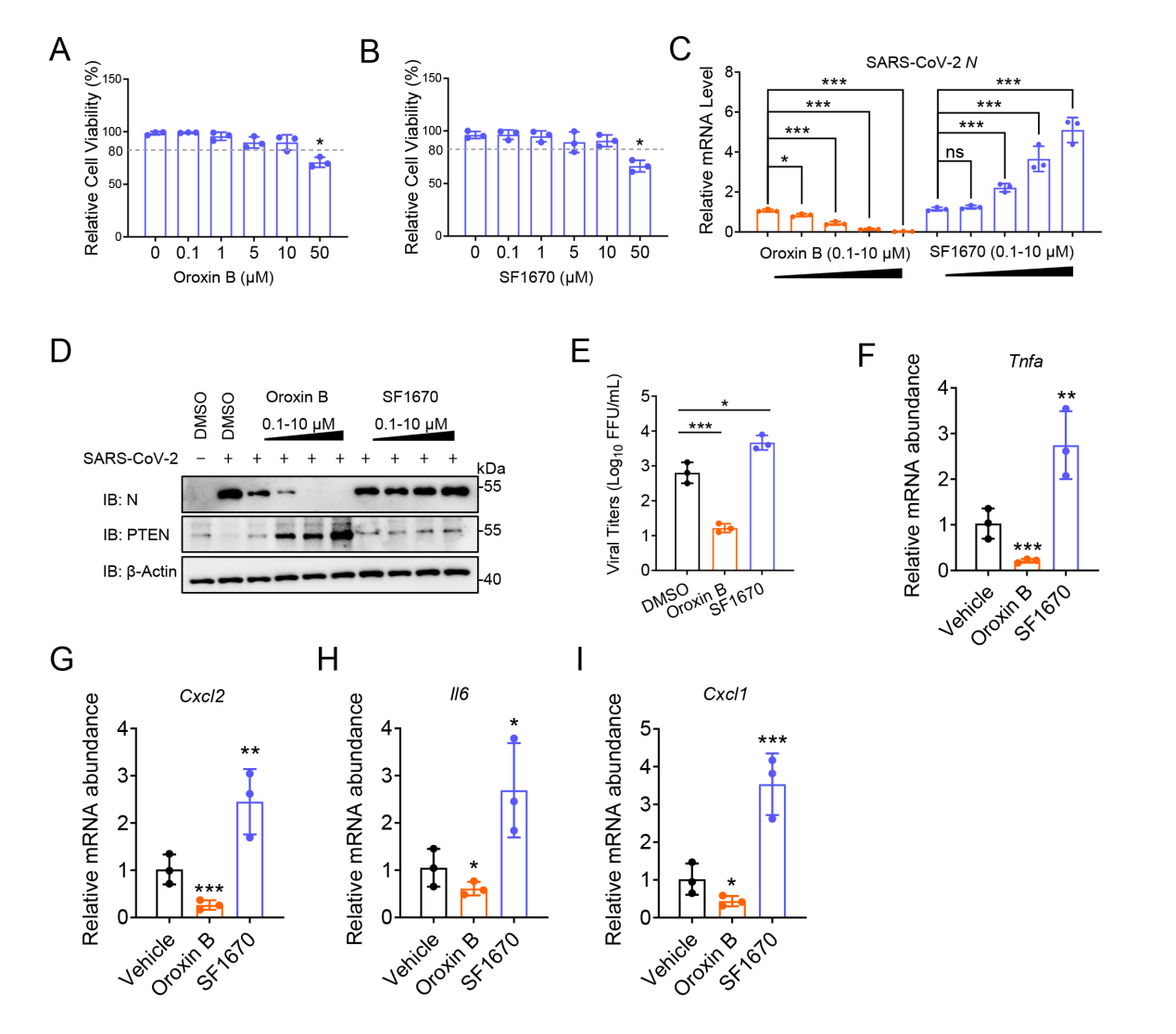
**

**Fig. S8 Oroxin B inhibits SARS-CoV-2 replication by enhancing PTEN expression.**

(A-B) After different concentrations of Oroxin B and SF1670 were added to Calu3 cells, Calu3 cell viability was assessed using a CCK-8 assay.

(C) Calu3 cells were inoculated in 24-well plates and treated with Oroxin B (0.1-10 µM) or SF1670 (0.1-10 µM) concurrently with SARS-CoV-2 infection (MOI=0.3). RNA was extracted from the cells 48 h later to detect the RNA level of the SARS-CoV-2 *N*.

(D) Calu3 cells were inoculated in 24-well plates and treated with Oroxin B (0.1-10 µM) or SF1670 (0.1-10 µM) concurrently with SARS-CoV-2 infection (MOI=1). Protein was extracted from the cells 48 h later to detect SARS-CoV-2 N protein levels.

(E) Calu3 cells treated with DMSO, Oroxin B, or SF1670 were infected with SARS-CoV-2 (MOI=0.3) for 24 h. Subsequently, the viral supernatant was used to infect Vero E6 cells for 0, 12, 24, 48 h with serial dilutions, followed by fixation with 4% paraformaldehyde, then staining with 0.1% crystal violet solution containing 1% paraformaldehyde. Error bars represent the SD of technical triplicates.

(F-I) Quantification of Tnfa, Cxcl2, Il6 and Cxcl1 mRNA levels in lung tissues from treated mice.

Data are expressed as mean ± SD (n = 3 independent experiments). * Indicates *P* < 0.05, ** indicates *P* < 0.01, and *** indicates *P* < 0.001, and statistical significance was determined using *Student's* *t-test*.

**Supplementary Table**

**Table S1:** The primers used for RT-qPCR were as follows:

| Gene name | Primers-F | Primers-R |
| --- | --- | --- |
| SARS-CoV-2 (*N*) | CGGAATGTCTCGCATCGGTA | GAGGAACGAGAAGAGGCTTG |
| *PTEN* | TGGATTCGACTTAGACTTGACCT | GCGGTGTCATAATGTCTCTCAG |
| Ifna | ACATGAGACCTTCAGCTCCAA | GGTCTGAGGACCTGAGGATGA |
| Ifnb | ATGACCAACAAGTGTCTCCTCC | GGAAGTTCGGTGACATCTCCC |
| Isg15 | CAGCCAGTCACTGTGAGAGG | GCTGCTGTGGAGGTGAAATA |
| Isg54 | CGAACTACCGTCTGGATGACTG | CTTCAACCAGCGCCATTGCTTG |
| Isg56 | GGCTTTGAGATTGTTCTGCTG | AAGCAGTTCTGGGTCAATGT |
| Tnfa | CAGGCGGTGCCTATGTCTC | CGATCACCCCGAAGTTCAGTAG |
| Il6 | TAGTCCTTCCTACCCCAATTTCC | TTGGTCCTTAGCCACTCCTTC |
| Cxcl2 | CATCCAGAGCTTGAGTGTGACG | GGCTTCAGGGTCAAGGCAAACT |
| Cxcl1 | TTGTTTCCACCGTAGCAGTC | TGTAGACCATGTAGTTGAGGTCA |
| *GAPDH* | GAAGGTGAAGGTCGGAGTCA | GGCTGTTGTCATACTTCTCATG |
